# Biosynthesis of insect sex pheromone precursors via engineered β-oxidation in yeast

**DOI:** 10.1093/femsyr/foac041

**Published:** 2022-08-10

**Authors:** Karolis Petkevicius, Leonie Wenning, Kanchana R Kildegaard, Christina Sinkwitz, Rune Smedegaard, Carina Holkenbrink, Irina Borodina

**Affiliations:** The Novo Nordisk Foundation Center for Biosustainability, Technical University of Denmark, Kemitorvet 220, 2800 Kgs. Lyngby, Denmark; BioPhero ApS, Lersø Parkallé 42-44, 4th, 2100 Copenhagen Ø, Denmark; BioPhero ApS, Lersø Parkallé 42-44, 4th, 2100 Copenhagen Ø, Denmark; BioPhero ApS, Lersø Parkallé 42-44, 4th, 2100 Copenhagen Ø, Denmark; BioPhero ApS, Lersø Parkallé 42-44, 4th, 2100 Copenhagen Ø, Denmark; BioPhero ApS, Lersø Parkallé 42-44, 4th, 2100 Copenhagen Ø, Denmark; BioPhero ApS, Lersø Parkallé 42-44, 4th, 2100 Copenhagen Ø, Denmark; The Novo Nordisk Foundation Center for Biosustainability, Technical University of Denmark, Kemitorvet 220, 2800 Kgs. Lyngby, Denmark; BioPhero ApS, Lersø Parkallé 42-44, 4th, 2100 Copenhagen Ø, Denmark

**Keywords:** β-oxidation, insect pheromones, *Yarrowia lipolytica*, fatty alcohols, fatty acids, peroxisomal oxidases

## Abstract

Mating disruption with insect sex pheromones is an attractive and environmentally friendly technique for pest management. Several Lepidoptera sex pheromones have been produced in yeast, where biosynthesis could be accomplished by the expression of fatty acyl-CoA desaturases and fatty acyl-CoA reductases. In this study, we aimed to develop yeast *Yarrowia lipolytica* cell factories for producing Lepidoptera pheromones which biosynthesis additionally requires β-oxidation, such as (*Z*)-7-dodecenol (*Z*7-12:OH), (*Z*)-9-dodecenol (*Z*9-12:OH), and (*Z*)-7-tetradecenol (*Z*7-14:OH). We expressed fatty acyl-CoA desaturases from *Drosophila melanogaster* (Dmd9) or *Lobesia botrana* (Lbo_PPTQ) and fatty acyl-CoA reductase from *Helicoverpa armigera* (HarFAR) in combinations with 11 peroxisomal oxidases of different origins. Yeast cultivations were performed with supplementation of methyl myristate (14:Me). The oxidase Lbo_31670 from *L. botrana* provided the highest titers of (*Z*)-7-dodecenoate, (*Z*)-9-dodecenoate, and (*Z*)-7-tetradecenoate. However, no chain-shortened fatty alcohols were produced. The mutation of fatty acid synthase (Fas2p^I1220F^) to increase myristate production did not lead to targeted fatty alcohol production. The problem was solved by directing the reductase into peroxisomes, where the strain with Dmd9 produced 0.10 ± 0.02 mg/l of *Z*7-12:OH and 0.48 ± 0.03 mg/l of *Z*7-14:OH, while the strain with Lbo_PPTQ produced 0.21 ± 0.03 mg/l of *Z*9-12:OH and 0.40 ± 0.07 mg/l of *Z*7-14:OH. In summary, the engineering of β-oxidation in *Y. lipolytica* allowed expanding the portfolio of microbially produced insect sex pheromones.

## Teaser

The authors engineered the yeast *Yarrowia lipolytica* for production of insect sex pheromones precursors.

## Introduction

Lepidoptera is the second largest order in the Insecta class and includes moths and butterflies (Wahlberg et al. 2013). Lepidopteran sex pheromones are fatty acid-derived metabolites that are biosynthesized and released by females to attract conspecific males for reproduction. Most of these pheromones are C_10_–C_18_ straight chain hydrocarbons with one to three double bonds and an oxygen-containing terminal group (alcohol, aldehyde, and alcohol acetate; Ando et al. 2004). The use of these oleochemicals is recognized as an environmentally friendly and effective method for pest control in agriculture. Their potential has been shown in several mating disruption studies, where the application of specific pheromone helped to reduce insect infestation (Alfaro et al. 2009, Hummel et al. 2015, Ioriatti and Lucchi 2016). Currently, chemical synthesis is the primary source of pheromones. However, in recent years, microbial and plant-based production has also been developed (Ding et al. 2014, Hagström et al. 2013a, Holkenbrink et al. 2020, Jiang et al. 2021, Petkevicius et al. 2021, Xia et al. 2020).

The fatty acid metabolism of yeast and plants can be engineered to enable the biosynthesis of pheromones and their precursors. Modifications needed to redirect common fatty acids, such as palmitate and stearate, toward pheromone biosynthesis include fatty acid desaturation, reduction, chain-shortening, alcohol acetylation, or oxidation (Petkevicius et al. 2020). A continuously growing list of sequenced insect genomes and transcriptomes facilitates enzyme discovery and characterization (Ding et al. 2014, 2021, Ding and Löfstedt 2015, Lassance et al. 2021). Fatty acyl-CoA desaturases (FADs) and fatty acyl-CoA reductases (FARs) are the most studied groups of enzymes related to pheromone biosynthesis. More than 50 FADs and 20 FARs from various insects have been characterized (Tupec et al. 2017). FADs introduce a double bond into a hydrocarbon chain while FARs are converting fatty acyl-CoAs into corresponding alcohols. Metabolic engineering efforts in yeast previously allowed to obtain *Saccharomyces cerevisiae* and *Yarrowia lipolytica* strains capable of producing insect pheromones and their precursors such as (*Z*)-9-tetradecenyl acetate (*Z*9-14:OAc), (*E*/*Z*)-11-tetradecenol (*E*/*Z*11-14:OH), (*Z*)-11-hexadecenol (*Z*11-16:OH), and (*Z*)-11-hexadecenal (*Z*11-16:Ald). Hagström et al. 2013b, Holkenbrink et al. 2020, Jiang et al. 2021, Petkevicius et al. 2021). The plants *Nicotiana benthamiana*, *N. tabacum*, and *Camelina sativa* have been used for production of ∆11 C_14_ and C_16_ fatty acid derivatives as well (Ding et al. 2014, Mateos-Fernández et al. 2021, Nešněrová et al. 2004, Ortiz et al. 2020). Additionally, a recent study in *C. sativa* demonstrated the production of a more challenging pheromone precursor of the codling moth *Cydia pomonella*-(*E*,*E*)-8,10-dodecadienoic acid (Xia et al. 2021). Our previous studies showed that strategies related to decreased degradation of fatty acids/alcohols and improved acyl-CoA supply are beneficial for the biosynthesis of C_14_ and C_16_ pheromones (Holkenbrink et al. 2020, Petkevicius et al. 2021).

Compared to fatty acid desaturation and reduction, chain-shortening, alcohol acetylation, and oxidation are underexplored, and information about enzymes performing these reactions in moths is scarce (Petkevicius et al. 2020). For the insects to produce pheromones of a specific carbon length, the oxidation of fatty acids needs to terminate after one to three β-oxidation cycles generating acyl-CoA of 10–14 carbons. This contrasts with the metabolic oxidation that proceeds to completion and generates acetyl-CoA. One β-oxidation cycle is composed of four reactions that take place in peroxisomes. Each cycle shortens the fatty acyl chain by two carbons and releases acetyl-CoA (Hiltunen et al. 2003). Peroxisomal oxidases (POXes) perform the first reaction converting acyl-CoA into trans-2-enoyl-CoA and hydrogen peroxide. The second and third steps are performed by a multifunctional enzyme (MFE2), which oxidizes trans-2-enoyl-CoA into 3-ketoacyl-CoA. In the final step, thiolytic cleavage catalyzed by 3-ketoacyl-CoA thiolase generates two-carbon shorter acyl-CoA and acetyl-CoA. POXes may vary in their chain length substrate specificities and, in this way, control the number of β-oxidation cycles (Luo et al. 2002). Multiple Lepidoptera POXes have been identified from genome and transcriptome sequencing data, however, until now, only several of them have been characterized (Antony et al. 2015, Chen et al. 2017, Ding et al. 2021, Dou et al. 2019).

While there are so far no reports in the literature on the production of pheromones by engineering peroxisomal β-oxidation, this engineering strategy has been applied for making some other fatty acid-derived chemicals. Oleaginous yeast *Y. lipolytica* and *Candida tropicalis* have been successfully engineered to produce fatty acid derivatives such as flavor lactones and adipic acid, respectively (Ju et al. 2020, Marella et al. 2020). In the case of *Y. lipolytica*, Marella et al. (2020) demonstrated that by replacing native POXes with a heterologous oxidase from *Rhinolophus sinicus* (RsAcox2), production of γ-dodecalactone from oleic acid could be increased 6-fold. RsAcox2 preferentially acted on acyl-CoAs of 14 carbons and above, and this resulted in the degradation of hydroxylated oleic acid to C_12_ precursor of γ-dodecalactone. Ju et al. (2020) showed that adipic acid production from methyl laurate could be increased by 5.4-fold when *C. tropicalis* broad-spectrum oxidase AOX4 was substituted by native oxidase AOX5, which was shown to have narrow substrate specificity (C_12_–C_10_). In some instances, it is beneficial to abolish β-oxidation and completely prevent fatty acid degradation. This was demonstrated by the biotechnology company Verdezyne, where *C. tropicalis* lacking POX4 and POX5 produced sebacic (C_10_) and dodecanedioic (C_12_) acids from the corresponding monocarboxylic acids without any significant degradation products (patent application number 201615272104). Such examples illustrate that β-oxidation can be engineered to enable biosynthesis of fatty acid derivatives with desired chain length.

**Figure 1.**
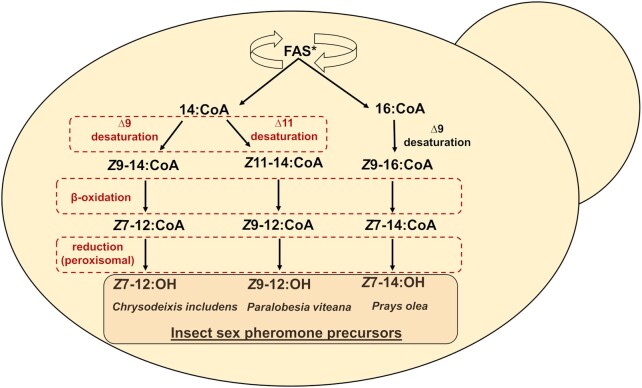
Schematic representation of metabolic pathways leading to three target insect sex pheromone precursors. Red color indicates steps, which were engineered in this study. FAS*: fatty acid synthase, where isoleucine was replaced to phenylalanine in the α chain (Fas2p^I1220F^) for increased production of 14:CoA.

In this study, we have selected three insect pheromone precursors as targets, namely, *Z*7-12:OH, *Z*9-12:OH, and*Z*7-14:OH, and constructed metabolic pathways towards them (Fig.   1). Acetate and aldehyde derivatives of these fatty alcohols are the main sex pheromone components of important pests, such as the soybean looper *Chrysodeixis includens*, the grape berry moth *Paralobesia viteana*, and the olive moth *Prays olea*, respectively (Campion et al. 1979, Roelofs et al. 1971, Tumlinson et al. 1972). We aimed to expand the portfolio of microbially produced insect sex pheromone precursors by engineered β-oxidation. Until now, this step was not implemented in the recombinant biosynthesis of insect pheromones, limiting the spectrum of possible products. To alleviate this bottleneck, we have chosen to work with the yeast *Y. lipolytica* due to its oleaginous properties and the availability of genetic engineering tools (Darvishi et al. 2018, Holkenbrink et al. 2018).

## Materials and methods

### Plasmid construction

Plasmid construction was performed based on the EasyCloneYALI toolbox described by Holkenbrink et al. (2018). Integrative and gRNA vectors were constructed and used to obtain chromosomal integration of expression cassettes in defined genomic loci of *Y. lipolytica*. Lists of primers, synthetic genes, biobricks, and plasmids used in this study are provided in Tables S1–S4 (Supporting Information), respectively. Biobricks were amplified by using Phusion U polymerase under the following conditions: 98°C for 5 min, 30 cycles of (98°C for 20 s, 54°C for 30 s, and 72°C for 30 s/kb), 72°C for 7 min. PCR products were purified using NucleoSpin Gel and PCR Clean-up kit (Macherey-Nagel). Assembly of plasmids was performed by USER^®^ cloning. Parent vectors were treated with FastDigest SfaAI (Thermo Fisher Scientific) restriction enzyme and Nb.BsmI (New England BioLabs) nicking endonuclease. Opened vector and compatible biobricks/primers were transformed into *Escherichia coli* strain DH5α. The correct assembly of plasmids was confirmed by colony PCR and Sanger sequencing.

### Yeast strain construction

Strains generated in this study were obtained using a lithium acetate-based transformation protocol described previously (Petkevicius et al. 2021). The list of strains is provided in Table S5 (Supporting Information). We used *Y. lipolytica* ST9138 as the background strain. The strain is derived from ST4840 (Y-17536), obtained from Agricultural Research Service (NRRL, USA). ST9138 has all six native POXes deleted (∆*pox1-6*), which provides a suitable background for testing different POX variants.

### Cultivation conditions

Yeast strains were inoculated from a YPD plate to an initial OD_600_ of 0.2 into 2.5 ml YPG medium (10 g/l yeast extract, 10 g/l peptone, and 40 g/l glycerol) in 24 well-plates with an air-penetrable lid (EnzyScreen; three replicates per strain). The plates were incubated at 28°C, shaken at 300 rpm at 5 cm orbit cast. After 22 h, the plates were centrifuged for 5 min at room temperature at 3000 × *g*. The supernatant was discarded, and the cells were resuspended in 1.25 ml production medium per well (50 g/l glycerol, 5 g/l yeast extract, 4 g/l KH_2_PO_4_, 1.5 g/l MgSO_4_, 0.2 g/l NaCl, 0.265 g/l CaCl_2_∙2H_2_O, and 2 ml/l trace elements solution: 4.5 g/l CaCl_2_∙2H2O, 4.5 g/l ZnSO_4_∙7H_2_O, 3 g/l FeSO_4_∙7H_2_O, 1 g/l H_3_BO_3_, 1g/l MnCl_2_∙4H_2_O, 0.4 g/l Na_2_MoO_4_, 0.3 g/l CoCl_2_∙6H_2_O, 0.1 g/l CuSO_4_∙5H_2_O, 0.1 g/l KI, and 15 g/l EDTA). Production medium was supplemented with methyl myristate (Sigma-Aldrich). Specific amounts are provided in the section of “Results and discussion.” The plates were incubated for 28 h at 28°C, shaken at 300 rpm.

### Sample preparation for fatty acid and alcohol analysis

For analysis of fatty acids, 500 µl of cultivation broth was transferred to 4 ml glass vials and centrifuged for 5 min at room temperature at 3000 ×*g*. The supernatant was decanted and the cell pellet exposed to transmethylation by 1 ml 1 M HCl in methanol (anhydrous). The samples were incubated at 70°C for 2 h. Every 30 min, the samples were vortexed for 10 s. After cooling down the samples to room temperature, 1 ml of 1 M NaOH in methanol (anhydrous), 500 µl of saturated NaCl solution in water, 500 µl of hexane, and 5 µl of methyl nonadecanoate (19:Me; 2 g/l) as internal standard were added. The samples were vortexed and centrifuged for 5 min at room temperature at 3000 × *g*. The upper organic phase was analyzed via gas chromatography–mass spectrometry (GC–MS).

For analysis of fatty alcohols, 500 µl of broth was transferred to 4 ml glass vials and centrifuged for 5 min at room temperature at 3000 × *g*. The pellet was treated with 500 µl of a mixture of ethyl acetate:ethanol (85:15, v/v) and 5 µl of 19:Me (2 g/l) was added as an internal standard. The samples were vortexed for 20 s and incubated for 1 h at room temperature followed by 5 min of vortexing. A volume of 300 µl of water were added, the samples were vortexed and centrifuged for 5 min at room temperature at 3000 × *g*. The upper organic phase was analyzed via GC–MS or gas chromatography–flame ionization detector (GC–FID).

### Analysis of fatty acid methyl esters and fatty alcohols by GC–MS and GC–FID

In this study, two gas chromatography systems were used-Agilent 7820A and Agilent 7890B. For fatty acid methyl esters (FAMEs) analysis, the Agilent 7820A system was coupled to a 5977B mass detector and equipped with a DB-Fatwax UI column (30 m × 250 μm × 0.25 μm). The operation parameters were: 1 μl injection, split ratio 20:1, injector temperature 220°C, constant flow 1 ml/min of helium, oven ramp 80°C for 1 min, 15°C/min to 210°C, 7 min hold time, and 20°C/min to 230°C. MS was scanning between m/z 30 and 350. The samples obtained from the supplementation assay (Fig. 3) were analyzed using previously described settings and temperature program (Petkevicius et al. 2021). The fatty alcohol profile shown in Fig. 4(A) was obtained as follows: all fatty alcohols except *Z*7-12:OH have been analyzed under the same conditions as FAMEs samples. *Z*7-12:OH has been identified and quantified using a HP-5 column (30 m × 320 μm × 0.25 µm) under the following conditions: 1 μl injection, split ratio 20:1, injector temperature 220°C, constant flow 1 ml/min of helium, oven ramp 80°C for 1 min, 15°C/min to 150°C, 7 min hold time, 10°C/min to 210°C, then 20°C/min to 300°C, and hold time for 5 min. MS was scanning between m/z 30 and 350. The chromatograms in Figures S1 and S2 (Supporting Information) were obtained by analyzing samples on a Agilent 7890B system equipped with a HP-5 column under the following conditions: 1 μl injection, split ratio 40:1, injector temperature 220°C, constant flow 30 ml/min of hydrogen, oven ramp 150°C for 3 min, 10°C/min to 210°C, and 20°C/min to 300°C. The quantification of compounds was performed based on the internal standard (19:Me) and the identity of compounds was confirmed based on comparison of retention times and mass spectra with reference standards. Reference standards were purchased from Pherobank.

### Fluorescence microscopy

Selected strains were cultivated as described in section “Cultivation conditions.” The cultivation broth was diluted 5-fold and 5 µl of the sample were loaded onto a microscope slide. Microscopy was performed using 100x magnification and immersion oil in a Leica DFC300 FX microscope equipped with a Leica EL600 external light source. Green fluorescence images were obtained using a GFP filter cube, while the red signal was obtained using a Y3 filter cube.

## Results and discussion

### Engineering β-oxidation in *Y. lipolytica* by replacement of native POXes with heterologous ones

We envisioned that screening multiple POX variants from different sources in the *∆pox1-6 Y. lipolytica* background strain would reveal the most suitable candidates to obtain fatty acids with the desired chain length and position of desaturation. More specifically, our goal was to find an oxidase that could efficiently convert the unsaturated C_14_ and C_16_ fatty acids [(*Z*)-9-tetradecenoic acid (*Z*9-14:acid), (*Z*)-11-tetradecenoic acid (*Z*11-14:acid), and (*Z*)-9-hexadecenoic acid (*Z*9-16:acid)] into two carbons shorter respective products: [(*Z*)-7-dodecenoic acid (*Z*7-12:acid), (*Z*)-9-dodecenoic acid (*Z*9-12:acid), and (*Z*)-7-tetradecenoic acid (*Z*7-14:acid)]. A total of 11 POX candidates from various sources were screened to select the most suitable POX. The list of POXes included three native *Y. lipolytica* oxidases, YliPOX2 (YALI0F10857g), YliPOX3 (YALI0D24750g), and YliPOX5 (YALI0C23859g), which are the major contributors to the overall peroxisomal acyl-CoA oxidase activity in this yeast (Wang et al. 1999). Additionally, POXes from *Aspergillus nidulans* (AniPOX), *Cucurbita maxima* (CmaPOX), *Homo sapiens* (HsaPOX), *Paenarthrobacter ureafaciens* (PurPOX), and *Rattus norvegicus* (RnoPOX) were selected due to their reported activities towards C_14_ and C_16_ acyl-CoAs (Bakke et al. 2007, Hayashi et al. 1998, Miyazawa et al. 1987, Oaxaca-Castillo et al. 2007, Reiser et al. 2010). Lastly, three oxidases from the insects *Agrotis segetum* (AsePOX) and *Lobesia botrana* (Lbo_31670, Lbo_49554) were included. In these moths, β-oxidation is postulated to be essential for producing the pheromone precursors, *Z*7-12:acid in the case of *A. segetum* and *Z*9-12:acid in the case of *L. botrana* (Ding and Löfstedt 2015, Ding et al. 2021).

POXes were screened in yeast strains either expressing Dmd9 FAD from *Drosophila melanogaster* or Lbo_PPTQ FAD from *L. botrana*, which provide the unsaturated precursors, *Z*9-14:CoA and *Z*11-14:CoA, respectively, while native *Y. lipolytica* desaturase YliOLE1 (YALI0C05951g) is responsible for the biosynthesis of *Z*9-16:CoA from 16:CoA.

In order to produce the target fatty alcohols, the FAR from *Helicoverpa armigera* (HarFAR) was considered a potentially suitable candidate. Previously, it was shown to act on a wide variety of fatty acyl-CoAs with a chain length ranging from C_8_ to C_16_ (Hagström et al. 2012). We rationalized that activity of the FADs and FAR in combination with different POXes could result in biosynthesis of *Z*7-12:OH, *Z*9-12:OH, and *Z*7-14:OH (Fig. 1).

**Figure 2.**
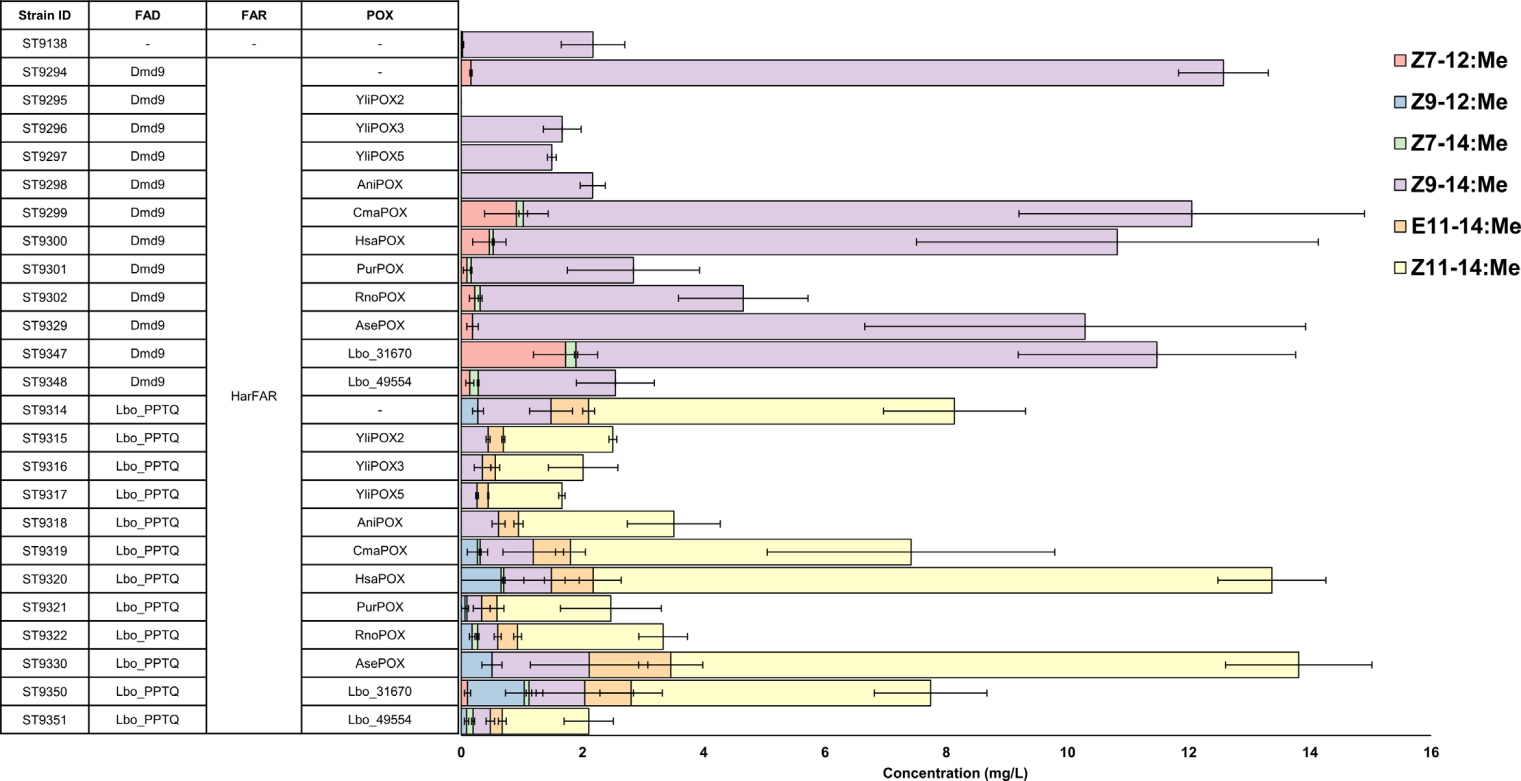
Profiles of C_12_ and C_14_ unsaturated fatty acids in the form of methyl esters obtained from the *Y. lipolytica* strains containing different FADs and POXes. Cultivation media was supplemented with 0.24% (v/v) of 14:Me. Error bars represent standard deviations from three technical replicates.

Strains containing different POXes combined with HarFAR and Dmd9 or Lbo_PPTQ did not produce any of the three target alcohols (Figures S1/S2, Supporting Information). Hence, we decided to evaluate the fatty acid profiles instead and determine if *Z*7-12:acid, *Z*9-12:acid, and *Z*7-14:acid are produced by yeast strains expressing different POXes (Fig. 2). The introduction of Dmd9 into parental strain ST9138 (∆*pox1-6*) resulted in a 6-fold increase in *Z*9-14:acid titer, reaching 12.4 ± 0.7 mg/l. Some background levels of this fatty acid in the parental strain could be explained by the endogenous activity of *Y. lipolytica* YliOLE1 desaturase. In the strain containing YliPOX2 no *Z*9-14:acid was detected. This oxidase was reported to have the highest activity towards 14:CoA among the set of tested fatty acyl-CoAs (8:CoA, 10:CoA, 12:CoA, 14:CoA, and 16:CoA), which is in agreement with the obtained results (Luo et al. 2002). The absence of *Z*7-12:acid suggests that YliPOX2 has a promiscuous activity, and β-oxidation does not stop after one cycle. The highest titer of *Z*7-12:acid was achieved with the oxidase Lbo_31670, which reached 1.72 ± 0.53 mg/l. The yeast strains expressing Dmd9 in combination with YliPOX3, YliPOX5, or AniPOX did not produce any *Z*7-12:acid, while the rest of the oxidases, CmaPOX, HsaPOX, PurPOX, RnoPOX, AsePOX, and Lbo_49554 generated between 0.09 and 0.91 mg/l of *Z*7-12:acid.

Introduction of Lbo_PPTQ (ST9314) yielded 6.0 ± 1.2 mg/l of *Z*11-14:acid and 0.6 ± 0.1 mg/l of (*E*)-11-tetradecenoic acid (*E*11-14:acid). Expression of Lbo_PTTQ was combined with expression of the individual POXes listed above and screened for production of *Z*9-12:acid. Production of Z9-12:acid was detected in strains expressing one of the POXes CmaPOX, HsaPOX, PurPOX, RnoPOX, AsePOX, Lbo_31670, or Lbo_49554, but not in strains expressing YliPOX2, YliPOX3, YliPOX5, or AniPOX. The strain with the highest production of *Z*9-12:acid, 0.93 ± 0.3 mg/l, combined the expression of the desaturase Lbo_PPTQ with the *L. botrana* oxidase Lbo_31670. Transcriptomics studies have shown that this POX is specifically expressed in the pheromone glands of *L. botrana* and contributes to the production of *Z*9-12:acid in this insect (Ding et al. 2021). A second POX from *L. botrana*, Lbo_49554, did not show strong expression bias towards pheromone glands, and produced approximately 10-fold lower amounts of *Z*9-12:acid than Lbo_31670 in this study. The differences between the two mentioned oxidases have been observed not only in the production levels of *Z*7-12:acid and *Z*9-12:acid, but also in the titers of (*Z*)-7-hexadecenoic acid (*Z*7-16:acid), which is the β-oxidation product of oleic acid (*Z*9-18:acid). In the set of strains expressing Dmd9, the level of *Z*7-16:acid was 3.7-fold higher in the Lbo_49554 expressing strain compared to the strain expressing Lbo_31670 (Figure S3, Supporting Information). A similar result was observed for the strains expressing Lbo_PPTQ, the difference was 3.1-fold (Figure S4, Supporting Information). This result supports the data obtained from transcriptomics studies and implies that Lbo_49554 is a metabolic oxidase while Lbo_31670 contributes to sex pheromone biosynthesis in *L. botrana*.

The highest titer of *Z*7-14:acid was achieved in the strain ST9347 (expresses Lbo_31670 POX) and reached 0.17 ± 0.03 mg/l. The ratio between the β-oxidation product (*Z*7-14:acid) and the precursor (*Z*9-16:acid) was 0.005, while in the best *Z*7-12:acid and *Z*9-12:acid producing strains (ST9347 and ST9350, respectively) the product/precursor ratios were approximately 36-fold higher. This shows that even the oxidase which provided the highest titer of *Z*7-14:acid among the tested variants is suboptimal, and in the future, more candidates could be screened for more efficient conversion of *Z*9-16:acid into *Z*7-14:acid.

In summary, fatty acid profiles revealed that strains containing Lbo_31670 POX provided the highest titers of *Z*7-12:acid, *Z*9-12:acid, and *Z*7-14:acid and the absence of corresponding alcohols most likely has other reasons than the lack of substrates.

### Validating activity of HarFAR on chain-shortened desaturated fatty acyl-CoAs

Since no target alcohols have been observed in the first round of strain screening, we have decided to test the ability of HarFAR to convert *Z*7-12:acid, *Z*9-12:acid, and *Z*7-14:acid into corresponding alcohols by supplying methyl esters of the acids mentioned above to the cultivation media as substrates. Previously, this reductase proved its versatility and has been used to produce various unsaturated fatty alcohols such as *Z*9-14:OH, *E*/*Z*11-14:OH, and *Z*11-16:OH. However, there was a lack of direct evidence if it can accept Z7-12:acid, Z9-12:acid, and Z7-14:acid as substrates (Holkenbrink et al. 2020, Petkevicius et al. 2021). Certain insect FARs are known to have very strict substrate specificities, such as reductases from *Ostrinia nubilalis*, while others have a broad substrate range (Tupec et al. 2017).

**Figure 3.**
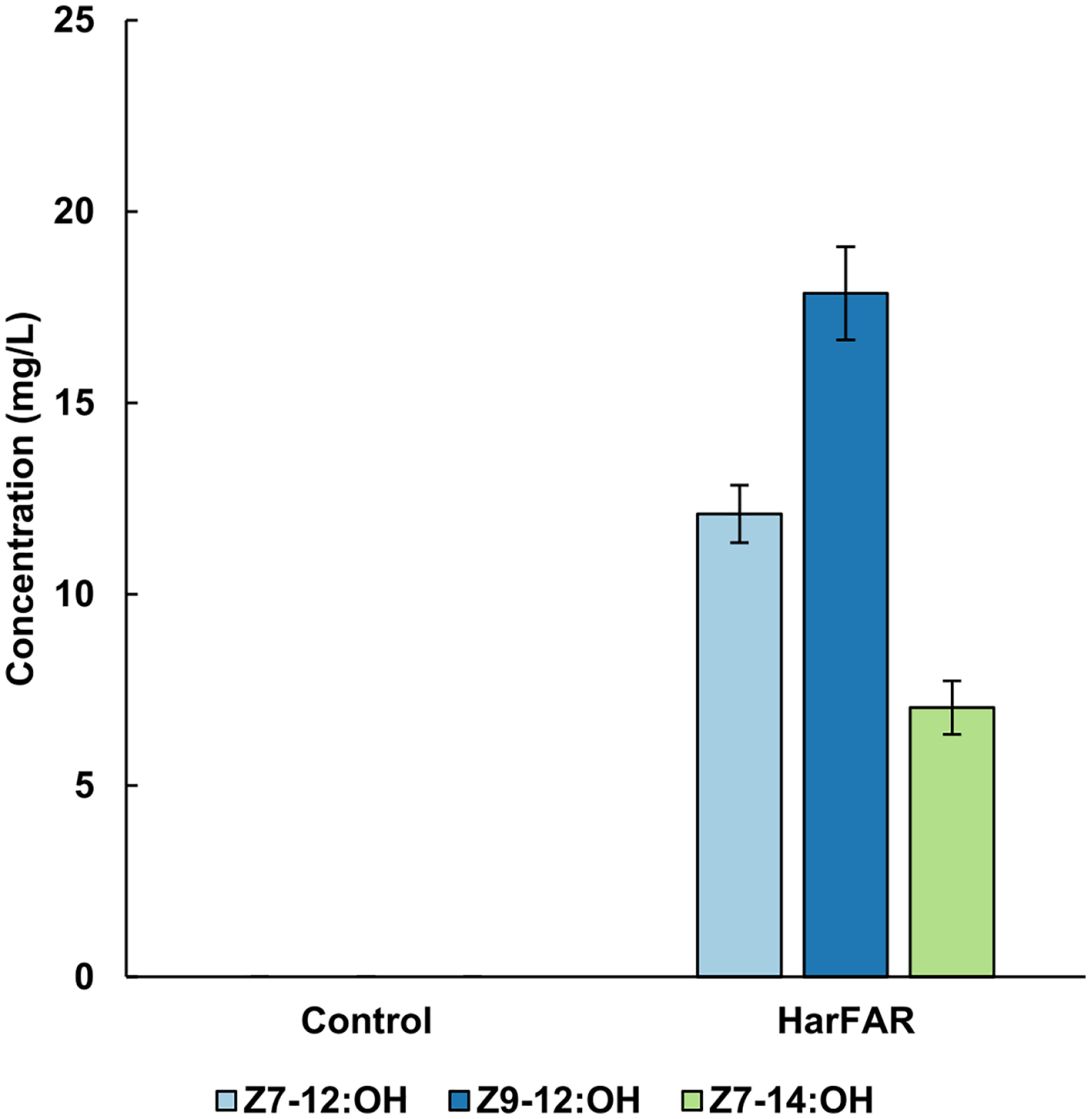
Production of *Z*7-12:OH, *Z*9-12:OH, and *Z*7-14:OH in the *Y. lipolytica* strain expressing HarFAR. Control is the strain lacking HarFAR. Cultivation media was supplemented with equal amounts (500 mg/l) of *Z*7-12:Me, *Z*9-12:Me, and *Z*7-14:Me. Error bars represent standard deviations from three technical replicates.

A *Y. lipolytica* strain expressing HarFAR under the strong constitutive *TEF1*intron promoter was used, and equal amounts of methyl esters of *Z*7-12:acid, *Z*9-12:acid, and *Z*7-14:acid were added to the culture medium (500 mg/l of each compound). Fatty alcohols were extracted from the cell pellet and subjected to GC–MS analysis. The strain expressing HarFAR converted supplied methyl esters into fatty alcohols while no production of these compounds was observed in the negative control strain without FAR. At the end of the cultivation, *Z*7-12:OH, *Z*9-12:OH, and *Z*7-14:OH reached titers of 12.1 ± 0.8 mg/l, 17.9 ± 1.2 mg/l, and 7.0 ± 0.7 mg/l, respectively, confirming that HarFAR is suitable for the biosynthesis of the three target compounds (Fig. 3).

### Enabling production of chain-shortened desaturated fatty alcohols by targeting the reductase into peroxisomes

**Figure 4.**
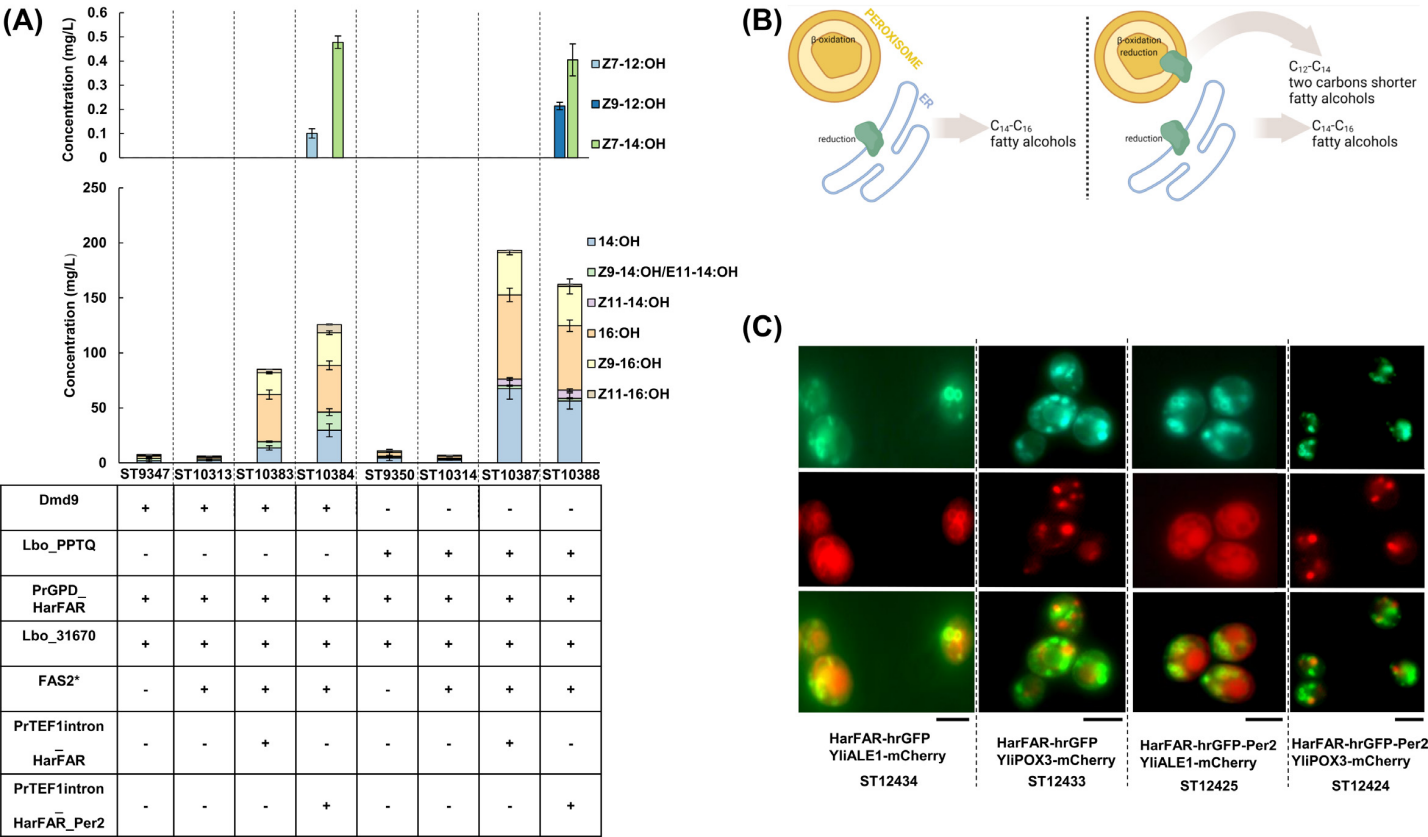
Production of *Z*7-12:OH, *Z*9-12:OH, and *Z*7-14:OH via peroxisomal targeting of HarFAR. (A) Fatty alcohol profiles of engineered *Y. lipolytica* strains. “−” and “+” indicate absence or presence of corresponding genetic change. In the strains expressing Dmd9, *Z*9-14:OH/*E*11-14:OH consists only of *Z*9-14:OH. Cultivation media was supplemented with 0.4% (v/v) of 14:Me. Error bars represent standard deviations from three technical replicates. (B) Schematic representation indicating coupling of β-oxidation and reduction by redirection of HarFAR (green) to peroxisomes. (C) Fluorescence microscopy images of strains coexpressing HarFAR fusion proteins (top row, green) and ER and peroxisomal markers (middle row, red). Bottom row shows overlay between the two images. Scale bars below the images correspond to 5 µm. FAS2*: modified α chain of fatty acid synthase (Fas2p^I1220F^).

The absence of the target fatty alcohols motivated us to improve the strains towards *in vivo* precursor supply and increased expression of HarFAR. Before increasing the expression of HarFAR, the best performing strains selected from the screening of POXes (ST9347 and ST9350) were mutated in the fatty acid synthase (FAS) α chain ketoacyl synthase domain. This resulted in ST10313 and ST10314, respectively. Replacement of isoleucine 1220 in Fas2p to phenylalanine (Fas2p^I1220F^) has been shown to increase myristic acid levels up to 8.4-fold (Petkevicius et al. 2021). Even though strains were engineered for increased myristic acid production, cultivation media was also supplemented with inexpensive 14:Me to ensure efficient precursor supply. Myristoyl-CoA is the precursor for production of *Z*7-12:OH and *Z*9-12:OH (Fig. 1). Fas2p^I1220F^ mutation did not increase C_14_ fatty alcohols titer, indicating that the reduction reaction and not precursor supply is limiting the flux towards the product (Fig. 4A). Next, an additional copy of HarFAR under the strong *TEF1*intron promoter was integrated into the genome of ST10313 and ST10314, resulting in strains ST10383 and ST10387, respectively. Compared to the GPD promoter, which was used for expression of HarFAR in ST10313 and ST10314, *TEF1*intron was reported to provide a 7-fold higher fluorescence signal when humanized Renilla Green Fluorescent Protein (hrGFP) was used as a reporter gene (Holkenbrink et al. 2018). For the expression of HarFAR under the *TEF1*intron promoter we have selected IntE_4 integration site located on chromosome E. Previously, a comparison of 12 integration sites showed that the highest expression of hrGFP is obtained from the IntE_4 site (Holkenbrink et al. 2018). This genome edit allowed to increase total fatty alcohol levels by 16.2-fold in the strain containing Dmd9 (ST10313 versus ST10383), while a 27.8-fold increase was observed in the case of Lbo_PPTQ expressing strain (ST10314 versus ST10387; Fig. 4A). Despite a significant increase in the total fatty alcohol titers, no production of *Z*7-12:OH, *Z*9-12:OH, or *Z*7-14:OH was detected.

This motivated us to explore the possibility of directing HarFAR into peroxisomes, which would allow β-oxidation and reduction reactions to occur in the same cellular compartment. In order to be shortened by two carbons, unsaturated acyl-CoAs have to access the lumen of peroxisomes and, after a β-oxidation cycle, travel back to the proximity of ER for reduction by FAR. We anticipated that the presence of HarFAR in peroxisomes would eliminate the need to export fatty acids out of peroxisomes and enable the production of the target fatty alcohols. To redirect HarFAR to the peroxisome, a 16-amino-acid-long peroxisomal targeting sequence peptide GGGSAAVKLSQAKSKL was C-terminally fused to HarFAR (HarFAR_Per2). This signal was used previously to target cytosolic FAR from *Marinobacter aquaeolei* (FaCoAR) into peroxisomes and helped to increase fatty alcohol production by 2.7-fold in *S. cerevisiae* compared to cytosolic FaCoAR (Zhou et al. 2016). Comparison between ST10383 and ST10384, both containing Dmd9, shows that HarFAR_Per2 improved total fatty alcohol titer by 19% compared to nonmodified HarFAR and enabled the production of *Z*7-12:OH and *Z*7-14:OH, which reached titers of 0.10 ± 0.02 mg/l and 0.48 ± 0.03 mg/l, respectively (Fig. 4A; Figure S5, Supporting Information). Among 14:OH, *Z*9-14:OH, 16:OH, *Z*9-16:OH, and *Z*11-16:OH, the biggest fold change was observed in *Z*9-14:OH, which increased by 3.2-fold. In general, the degree of unsaturation (calculated as the ratio between the sum of unsaturated fatty alcohols and total fatty alcohols) was higher in ST10384 (0.43 ± 0.01) compared to ST10383 (0.34 ± 0.01). The same trend was observed previously when this parameter was compared between the *S. cerevisiae*-produced fatty alcohols by the action of cytosolic and peroxisomal FaCoAR (Zhou et al. 2016). Expression of HarFAR_Per2 in the strain containing Lbo_PPTQ enabled the production of *Z*9-12:OH, and *Z*7-14:OH, and titers reached 0.21 ± 0.03 mg/l and 0.40 ± 0.07 mg/l, respectively (Fig. 4A; Figure S6, Supporting Information). These compounds were not produced in the strain containing nonmodified HarFAR (ST10387).

Interestingly, the strains containing Lbo_PPTQ had higher total fatty alcohol levels than respective Dmd9 strains (ST10383 versus ST10387 and ST10384 versus ST10388). Additionally, HarFAR_Per2 did not provide higher total fatty alcohol levels, while the degree of unsaturation was still higher (0.25 ± 0.01 in ST10387 and 0.30 ± 0.05 in ST10388). The differences in total fatty alcohol levels between the respective Dmd9 and Lbo_PPTQ expressing strains could be related to the fatty acid levels in the parent strains [Figure S3 (Supporting Information) ST9347, Figure S4 (Supporting Information) ST9350]. ST9350 had 37.2% higher total FAMEs content compared to ST9347. It was previously reported that the expression of heterologous desaturases increases lipid production in *Y. lipolytica*, possibly through alleviating feedback inhibition caused by saturated fatty acyl-CoAs (Yan et al. 2020). It could be hypothesized that different unsaturated fatty acids have distinct effects on the FAS complex to modulate the fatty acid profile differently. In addition to deep-well cultivations, shake flask cultivations were performed (Figure S7, Supporting Information), and production of the target fatty alcohols reconfirmed. The titers and specific yields can be found in Table S6 (Supporting Information).

Previously, insect sex pheromone alcohols such as *Z*11-16:OH, *Z*9-14:OH, and *Z*11-14:OH have been microbially produced in *S. cerevisiae* and *Y. lipolytica*. In most of these studies, production of unsaturated fatty alcohols was achieved by expression of FADs and FARs. Expression of FAD and FAR from turnip moth (*A. segetum*) in *S. cerevisiae* enabled the production of *Z*11-16:OH at 0.195 mg/l (Hagström et al. 2013a). The same compound was later produced in the yeast *Y. lipolytica* by expressing multiple copies of Atr∆11 FAD from *Amyelois transitella* together with FARs from *Helicoverpa* spp. Additionally, modifications preventing fatty alcohol degradation (∆*FAO1*), fatty acid degradation (∆*PEX10*), and triacylglycerol formation (Pr_100_GPAT) were combined resulting in the strain capable of producing 2.57 g/l of *Z*11-16:OH. In the same study, combining FAD from *D. melanogaster* with HarFAR and *FAS2* mutation (Fas2p^I1220F^) yielded 73.6 mg/l *Z*9-14OH (Holkenbrink et al. 2020). *Z*11-14:OH reached the titer of 188.1 mg/l in *Y. lipolytica*, where Lbo_PPTQ desaturase and HarFAR reductase served as enzymes converting 14:CoA into Z11-14:OH (Petkevicius et al. 2021). Recently, a different approach was demonstrated by Jiang et al. (2021). Instead of FARs, which use acyl-CoAs as substrates for fatty alcohol formation, the authors employed carboxylic acid reductase (CAR) that acts on free fatty acids and results in aldehyde production. Use of FAD from *H. armigera* in combination with CAR from *Mycobacterium marinum* enabled biosynthesis of (*Z*)-11-hexadecenal (*Z*11-16:Ald) at the level of 22.7 mg/l (Jiang et al. 2021). The present study employed yet another element-peroxisomal β-oxidation to expand the list of microbially produced insect sex pheromone alcohols.

Chemically, the synthesis of target fatty alcohols (*Z*7-12:OH, *Z*9-12:OH, and *Z*7-14:OH) could be accomplished by cross-metathesis where α,ω-diols and α-olefins serve as starting materials (patent application number 20200039900). In order to obtain the desired isomer, complex metal catalysts ensuring correct double bond configuration have to be used (Quigley and Grubbs 2014). In contrast, bio-based production employs renewable feedstocks, such as glycerol or glucose, and the specificity of desaturases ensures the desired stereochemistry.

In order to visually inspect the cellular localization of HarFAR and HarFAR_Per2, HarFAR was C-terminally tagged with hrGFP or hrGFP_Per2. The yeast strains were additionally transformed with either mCherry-tagged YliALE1 (YALI0F19514g) or YliPOX3, serving as ER membrane and peroxisomal marker proteins, respectively (Fig. 4C). In the strains expressing HarFAR-hrGFP (ST12434 and ST12433), a distinct circular green signal was obtained, i.e. typical for ER membrane proteins. This signal colocalized with YliALE1-mCherry signal (ST12434) but not with YliPOX3-mCherry (ST12433), indicating that HarFAR is present in the ER membrane, but not in peroxisomes. This result is in agreement with previous studies that have shown that FARs from mouse or noctuidae family of insects are ER membrane resident proteins (d’Espaux et al. 2017, Hagström et al. 2013b). Strains expressing HarFAR-hrGFP-Per2 showed a pattern of small green granules distinct from ER signal (ST12425), and in certain places overlaid with the punctate signal from YliPOX3-mCherry (ST12424), demonstrating peroxisomal localization of reductase tagged with hrGFP_Per2. However, redirection from ER to peroxisomes appeared to be suboptimal, since a green signal was also observed in the locations where YliPOX3-mCherry signal was absent. SKL tripeptide at the C-terminus of Per2 sequence is known as canonical peroxisomal targeting sequence 1 (PTS1). This sequence is recognized by Pex5p cytosolic receptor, which guides SKL-containing proteins into peroxisomes (Walter 2019). Apart from PTS1, peroxisomal targeting sequence 2 (PTS2) can also direct proteins to peroxisomes. PTS2 is located at N-terminus and has the following sequence: (R,K)-(L,V,I)-X_5_-(H,Q)-(L,A,F). This signal is recognized by Pex7p, which carries proteins to peroxisomes (Schafer et al. 2004). Interestingly, the study by Rosenthal et al. (2020) showed that Pex5p cargo proteins have different targeting priorities. Proteins such as Cat2p and Lys2p have been shown to have high targeting priority. Targeting priority turned out to be related to the high binding affinity of those proteins’ last 10 amino acids to Pex5p (Rosenthal et al. 2020). Alternative designs could be explored in the future for more efficient HarFAR targeting. For example, instead of Per2, C-terminal sequences from Cat2p or Lys2p could be used or/and effects of PTS2 signal could be explored. Furthermore, overexpression of PEX5 and PEX7 might improve targeting since those two receptors are involved in protein trafficking to peroxisomes.

Both fusion proteins (HarFAR-hrGFP and HarFAR-hrGFP-Per2) were enzymatically active and generated around 350 mg/l of fatty alcohols (Figure S8, Supporting Information). Interestingly, apart from the typical ER pattern, HarFAR-hrGFP also provided bright, dotted signals that were distinct from ER and peroxisomal signals. Previously, it was shown that proteins with predicted transmembrane domains and ER localization can also be found in lipid droplets. This was experimentally proven for *Y. lipolytica* lipid-modifying enzymes such as DGA1 (YALI0E32769g) and SLC1 (YALI0E18964g; Bredeweg et al. 2017). Lipid droplets are generally believed to be derivatives of the ER, where synthesized neutral lipids aggregate with ER membrane and form separate cellular compartments surrounded by a membrane monolayer (Jacquier et al. 2011). Studies in *S. cerevisiae* show that lipid droplet formation is initiated at specific ER regions which contain Fld1 and Nem1 proteins. Fld1-Nem1 site recruits TAG-producing enzymes such as Lro1 or Dga1, leading to neutral lipid synthesis and lipid body growth and maturation (Choudhary et al. 2020).

## Conclusions

In this study, we have established bioproduction of insect sex pheromone alcohols, namely, *Z*7-12:OH, *Z*9-12:OH, and *Z*7-14:OH. This was achieved by engineering β-oxidation, where the native *Y. lipolytica* POXes were replaced by the *L. botrana* oxidase Lbo_31670. Unsaturated fatty acid precursors were generated by the action of FADs, Dmd9, Lbo_PPTQ, and YliOLE1, while peroxisomally expressed FAR was used for fatty alcohol formation. The work paves the way toward a broader spectrum of biologically produced insect sex pheromone components.

## Authors’ contributions

K.P., I.B., and C.H. conceived and designed the study. L.W. and K.R.K. participated in molecular biology work (plasmid design and construction, sequencing analysis, and so on), C.S. and R.S. participated in the GC–MS analysis. K.P. performed the experiments, analyzed the data, and drafted the manuscript. I.B. and C.H. edited and proof-read the manuscript. All authors read and approved the final manuscript.

## Acknowledgments

The authors acknowledge Dr. Lyubomir Stanchev for discussions and advices regarding fluorescence microscopy experiment.

## Supplementary Material

foac041_Supplemental_File
