# Supplementary material for: Biosynthesis of insect sex pheromone precursors via engineered β-oxidation in yeast
Source: FEMS Yeast Res. 2022 Aug 10;22(1):foac041. doi: 10.1093/femsyr/foac041 (PMC9435373; doi:10.1093/femsyr/foac041)
Supplement: foac041_Supplemental_File [file foac041_supplemental_file.pdf]

Supplementary material for the research article

Biosynthesis of insect sex pheromone precursors via engineered β-oxidation in yeast

Karolis Petkevicius[1,2], Leonie Wenning[2], Kanchana Rueksomtawin Kildegaard[2], Christina Sinkwitz[2], Rune Smedegaard[2], Carina Holkenbrink[2], Irina Borodina[1,2,*]

[1]The Novo Nordisk Foundation Center for Biosustainability, Technical University of Denmark, Kemitorvet 220, 2800 Kgs. Lyngby, Denmark

[2]BioPhero ApS, Lersø Parkallé 42-44, 4th, 2100 Copenhagen Ø, Denmark

*Correspondence: Irina Borodina

Email: irbo@biosustain.dtu.dk

Supplementary table S1. Primers used in this study

ID	Description	Sequence 5' to 3'
PR-18928	PrTEF1intron <-_U1_fw	CACGCGAU AGAGACCGGGTTGG
PR-18975	<-PrTEF1_fw	ACCTGCACUTTTGAATGATTCTTATAC
PR-18066	Har_FAR_U2_fw	ATCTGTCAUGCCACAATGGTGGTCCTGACCTCTAAG
PR-16595	Har_FAR_codoptYL_U2_rev	CACGCGAUCTACTCGTAGGACTTCTTCTC
PR-18930	PrTEF1intron <-_forfusion_U1_fw	ATCAGTAGCU AGAGACCGGGTTGG
PR-18214	PrTEF1intron_USER_rv	AGTACTGCAAAAAGUGCTG
PR-23004	PrGPD_forfusion_U1_fw	ATCAGTAGCUGACGCAGTAGGATGTCCTGC
PR-22213	PrGAPHD->_U2_rev	ATGACAGAU TGTTGATGTGTGTTTAATTCAAGAATG
PR-19018	Dmd9_U1_rev	CGTGCGAUTTATCGAGACTTGTCC
PR-21723	<-Lbo_PPTQ_U1_fw	ACTTTTTGCAGTACUAACCGCAG GTGCCTCGAGCCGCTTCGG
PR-21724	<-Lbo_PPTQ_U1_rev	CGTGCGAU TTACTCCTTCTTAGCGTG
PR-23435	Ase_POX_U1_fw	AGTGCAGGUGCCACA ATGCCCATCTTCATCTG
PR-23436	Ase_POX_U1_rv	CGTGCGAUTTACAGCTTAGACTGCA
PR-21755	Lbo31670->_U2_fw	ATCTGTCAU GCCACA ATGACTGAGGTGACCAAGG
PR-21756	Lbo31670->_U2_rev	CACGCGAU TTACAGCTTACCCTGCATG
PR-15521	PrExp_fw	CGTGCGAUAAGGAGTTTGGCGCCCGTT
PR-15522	PrExp_rev	ATGACAGAUTGCTGTAGATATGTCTTGT
PR-21757	Lbo49554->_U2_fw	ATCTGTCAU GCCACA ATGGAGTCTAAGGACTTTG
PR-21758	Lbo49554->_U2_rev	CACGCGAUTTACAGCTTGGCGGGCACG
PR-22827	Yli_POX2_fw	AGTGCAGGUGCCACAATGAACCCC
PR-22828	Yli_POX2_rv	CGTGCGAUCTATTCCTCATCAAG
PR-22829	Yli_POX3_fw	AGTGCAGGUGCCACAATGATCTCCC
PR-22830	Yli_POX3_rv	CGTGCGAUCTATTCCTCGTCCAG

PR-22833	Yli_POX5_fw	AGTGCAGGUGCCACAATGAACAACAAC
PR-22834	Yli_POX5_rv	CGTGCGAUCTACTCGTCCAGGTC
PR-22841	Ani_POX_fw	AGTGCAGGUGCCACAATGCCCAACC
PR-22842	Ani_POX_rv	CGTGCGAUCTACAGCTTAGACTTG
PR-22843	Cma_POX_fw	AGTGCAGGUGCCACAATGGCCGCTG
PR-22844	Cma_POX_rv	CGTGCGAUCTACAGCTTAGACGAAG
PR-22845	Hsa_POX1-2_fw	AGTGCAGGUGCCACAATGAACCCCG
PR-22846	Hsa_POX1-2_rv	CGTGCGAUCTACAGCTTCGAAGAAG
PR-22847	Pur_POX_fw	AGTGCAGGUGCCACAATGACCGAGG
PR-22848	Pur_POX_rv	CGTGCGAUCTACAGCTTCGAAGAAG
PR-22849	Rno_POX-2_fw	AGTGCAGGUGCCACAATGAACCCCG
PR-22850	Rno_POX-2_rv	CGTGCGAUCTACAGCTTCGAAGAAG
PR-10595	PrTEF1intron _fw	CGTGCGAUAGAGACCGGGTTGG
PR-24919	HarFAR_Per2_U2_rev	CACGCGAUCTAAAGCTTAGACTTGGCCTGAGAAAGCTT AACGGCGGCGGAGCCTCCGCCCTCGTAGGACTTCTTC
PR-23172	IntD2_dwn_fwd	AGTGGCCUGACCAACCTTGTTTGG
PR-23173	IntD2_dwn_rev	CACGCGAUGCATATACGATTTGACTG
PR-23171	IntD2_up_fwd	CGTGCGAUCGTTTCGGAATGTGTC
PR-23170	IntD2_up_rev	AAGCGTTGCACGUTAAGTTGAGAGAGAACGC
PR-23167	IntE_7_up_fwd	CGTGCGAUGAATCTTGGTGCTCAAC
PR-23166	IntE_7_up_rev	AAGCGTTGCACGUGCAATCCGAAGAAGC
PR-23168	IntE_7_dwn_fwd	AGTGGCCUTACTCACATCAGATGGTC
PR-23169	IntE_7_dwn_rev	CACGCGAUGACACATGTGTCTACG
PR-22532	IntF_5_up_fwd	CGTGCGAUGAAGGCTACAACAAGGG
PR-22533	IntF_5_up_rev	AAGCGTTGCACGUTTTTTACATCACGTGCC
PR-22534	IntF_5_down_fwd	AGTGGCCUTGCGCTCACTCGTGATG
PR-22535	IntF_5_down_rev	CACGCGAUCTTGTTTCCCATAGTTTAATG
PR-26919	HarFARforhrGFP_rv	ACCACCCTCGUAGGACTTCTTCTC
PR-26920	hrGFPforHarFAR_fw	ACGAGGGTGGUGGTTCTGTGAGCAAGCAGATC
PR-15506	hrGFP_U2_rev	CACGCGAUTTACACCCACTCGTGCA
PR-26921	hrGFPPer2_rv	CACGCGAUTTAAAGCTTAGACTTGGCCTGAGAAAGCTTAACGG CGGCGGAGCCTCCGCCCACCCACTCGTGCAG
PR-24936	POX3 Y.l. for TEF1intron fw	ACTTTTTGCAGTACUAACCGCAGATCTCCCCCAACCTC
PR-26932	YliPOX3formCherry_rv	ACCACCTUCCTCGTCCAGCTC
PR-26926	YliALE1forTEF1intron_fw	ACTTTTTGCAGTACUAACCGCAGGCCTTTCCATGG
PR-26933	YliALE1formCherry_rv	ACCACCCTUGGTCTTGATGG
PR-26934	mCherryforYliPOX3_fw	AAGGTGGUGGTTCTGTGTCTAAGGGCGAAG
PR-26936	mCherry_U2_rv	CACGCGAUCTACTTGTACAGCTC
PR-26935	mCherryforYliALE1_fw	AAGGGTGGUGGTTCTGTGTCTAAGGGCGAAG

Supplementary table S2. Synthetic genes used in this study

ID	Gene	Sequence 5' to 3'
Sequence No. 1	Fatty acyl reductase from *Helicoverpa armigera*	ATGGTGGTCCTGACCTCTAAGGAGACTAAGCCCTCCGTGGCCGAGTTCTACGCTGGCAAGTCTGT CTTCATCACCGGCGGAACCGGTTTCCTGGGCAAGGTCTTCATTGAGAAGCTGCTGTACTCCTGTC CCGACATCGGCAACATCTACATGCTGATCCGAGAGAAGAAGGGACTGTCTGTGTCCGAGCGAATT AAGCACTTCCTGGACGACCCCCTGTTCACCCGACTGAAGGAGAAGCGACCCGCCGACCTGGAGAA GATCGTGCTGATTCCCGGAGACATCACCGCTCCCGACCTGGGTATTACCTCTGAGAACGAGAAGA TGCTGATCGAGAAGGTGTCTGTCATCATTCACTCCGCCGCTACCGTCAAGTTCAACGAGCCCCTG

		CCCACCGCCTGGAAGATCAACGTGGAGGGAACCCGAATGATGCTGGCTCTGTCTCGACGAATGAA<br>GCGAATTGAGGTCTTCATCCACATTTCCACCGCCTACACCAACACCAACCGAGAGGTGGTGGACG<br>AGATCCTGTACCCTGCTCCTGCTGACATTGACCAGGTGCACCGATACGTCAAGGACGGTATCTCT<br>GAGGAAGAGACTGAGAAGATTCTGAACGGCCGACCCAACACCTACACCTTCACCAAGGCCCTGAC<br>CGAGCACCTGGTGGCTGAGAACCAGGCTTACGTGCCCACCATCATTGTCCGACCCTCCGTGGTCGC<br>CGCTATCAAGGACGAGCCCATTAAGGGATGGCTGGGTAACTGGTACGGAGCTACCGGACTGACCG<br>TGTTCACCGCTAAGGGTCTGAACCGAGTCATCTACGGCCACTCTTCCAACATCGTGGACCTGATT<br>CCCGTGGACTACGTCGCCAACCTGGTCATTGCCGCTGGCGCTAAGTCTTCCAAGTCCACCGAGCTG<br>AAGGTGTACAACTGTTGCTCTTCCGCCTGCAACCCCATCACCATTGGAAAGCTGATGTCTATGTT<br>CGCCGAGGACGCTATCAAGCAGAAGTCCTACGCTATGCCCCTGCCCGGTTGGTACATCTTCACCA<br>AGTACAAGTGGCTGGTCCTGCTGCTGACCATTCTGTTCCAGGTCATCCCCGCCTACATTACCGACC<br>TGTACCGACACCTGATCGGCAAGAACCCCCGATACATTAAGCTGCAGTCTCTGGTCAACCAGACC<br>CGATCTTCCATTGACTTCTTCACCTCTCACTCCTGGGTCATGAAGGCTGACCGAGTCCGAGAGCT<br>GTTCGCCTCTCTGTCCCCCGCTGACAAGTACCTGTTCCCCTGTGACCCCACCGACATCAACTGGAC<br>CCACTACATTCAGGACTACTGCTGGGGAGTGCGACACTTCCTGGAGAAGAAGTCCTACGAGTAG
Sequence No. 2	Fatty acyl desaturase from *Drosophila melanogaster*	ATGGCTCCCTACTCTCGAATCTACCACCAGGACAAGTCGTCCCGAGAGACTGGCGTGCTGTTCGA<br>GGACGACGCCCAGACCGTGGACTCTGACCTGACCACCGACCGATTCCAGCTGAAGCGAGCCGAGA<br>AGCGACGACTGCCCCTGGTGTGGCGAAACATCATCCTGTTCGCCCTGGTGCACCTGGCCGCTCTGT<br>ACGGCCTGCACTCTATCTTCACCCGAGCCAAGCTGGCCACCACTCTGTTCGCTGCCGGCCTGTACA<br>TCATCGGCATGCTGGGCGTGACCGCTGGCGCCCACCGACTGTGGGCTCACCGAACCTACAAGGCC<br>AAGTGGCCCCTGCGACTGCTGCTGGTGATCTTCAACACCATTGCCTTCCAGGACGCCGTGTACCAC<br>TGGGCCCGAGATCACCGAGTGCACCACAAGTACTCTGAGACTGACGCTGACCCTCACAACGCTAC<br>CCGAGGCTTCTTCTTCTCTCACGTCGGCTGGCTGCTGTGCAAGAAGCACCCCGACATCAAGGAAA<br>AGGGCCGAGGCCTGGACCTGTCTGACCTGCGAGCTGACCCCATCCTGATGTTCCAGCGAAAGCAC<br>TACTACATTCTGATGCCCCTGGCCTGCTTCGTGCTGCCCACCGTGATTCCCATGGTGTACTGGAAC<br>GAGACTCTGGCCTCTTCCTGGTTCGTGGCCACCATGTTCCGATGGTGCTTCCAGCTCAACATGACC<br>TGGCTGGTGAACTCTGCCGCTCACAAGTTCGGCAACCGACCTTACGACAAGACTATGAACCCCAC<br>TCAGAACGCCTTCGTGTCTGCCTTCACCTTCGGCGAAGGCTGGCACAACTACCACCACGCATTCCC<br>TTGGGACTACAAGACCGCCGAGTGGGGCTGCTACTCTCTGAACATCACCACCGCCTTCATCGACC<br>TGTTCGCTAAGATCGGCTGGGCCTACGACCTCAAGACCGTGGCTCCCGACGTGATCCAGCGACGA<br>GTGCTGCGAACCGGCGACGGCTCTCACGAGCTGTGGGGCTGGGGCGACAAGGACCTGACCGCTGA<br>GGACGCCCGAAACGTCCTGCTGGTGGACAAGTCTCGATAA
Sequence No. 3	Fatty acyl desaturase from *Lobesia botrana*	ATGGTGCCTCGAGCCGCTTCGGAGGAGACCGACCTTAAGGAGGCTACCCAGCTTGAGCCCCGAAA<br>GTACGAGATCGTGTACACTAACGTGATCTACTTCACCTATTGGCATATCGCCGGACTGTACGGTC<br>TGTACCTGTGTTTTACCTCCGCTAAATGGGAGACCATCGTGTTCGCTTGGGCTTGGTATGTGCTC<br>GGAGAGCTGGGAGTGATTGCCGGCGCTCATAGATTGTGGGCCCACCGAACCTACAAGGCAAAGAT<br>GCCCCTGCAGATCATCCTGATGCTGTTTAATTGTATCGGTTTTCAGAACACCGCTACCGATTGGG<br>TTCGAGATCACCGAGTGCATCACAAGCACTCTGACACCGACGCCGACCCCCATAACTCTCAGCGA<br>GGCTTCTTTTTCTCTCACGTGGGCTGGCTGCTGACCCGAAAGCATCGGCTGGTGAAGGAGAAGGG<br>AGAAGCTGTTGACATGACTGATATCTACTCTAACCCTGTTTTAAGATTCCAGAAGAAGTACTCTC<br>TCCCCCTGATCGGCACTCTTTGCTTTGCTCTGCCCACCCTGCTGCCCGCTTACTGCTGGGGAGAGG<br>CCGTCGGCACCGCTTGGAACATTAACCTGCTGCGATACTGTCTTAACCTGAACGGAACCTTCCTG<br>ACTAACTCCGCCGCTCACAAGTTTGGCTCTAAGCCCTATGACAAGACCATTCCTCCCACCCAGAAC<br>TTGCTGGTGTCTTTCATGACTCTGGGAGAAGGATTTCATAATTACCACCACGTCTTCTCGTGGGA<br>CTACCGAGCTGCTGAGCTTGGCAACACCTACCTGAACATGACCACTATCTTTATCGACTTCTTCG<br>CTCTTATTGGATGGGCCTACGACCTGAAGACCGTTCCTGAGGATGTTATTAAAAAGCGAATGGCC<br>CGAACTGGAGATGGTACTAACCTGTGGGGTTGGGGAGACAAGGACATGACCAAGGAGGACGTGG<br>TGGACACCGAGATACGATTCCACGCTAAGAAGGAGTAA
Sequence No. 4	Peroxisomal oxidase from *Agrotis segetum*	ATGCCCATCTTCATCTGCATCATCACCTCTCAGGCCATCATCCGATCTAACGTCGAGCGAGTGGCC<br>GTGATCCTGAACATCAACATGGGCAAGGTGAACGAGGACCTGGTGCGAGAGCGAGCCAAGTGCAC<br>CTTCAACATCGAGGAACTGACCTACTTCCTGGACGGCGGCAAGGACAAGACCCTCGAGCGAAAGG<br>AAACCGAGCGAGCTATGCTGACCAAGCGAGAGGAACTGTTCGGCGGCGTGCCCGACGAGTACCTG<br>TCTCACAAGGAAAAGTACGAGAACTCTATGCGAAAGGCCGTGATTCTGTTCGGCATCCTGCGAAA<br>GATCCAGAAGGACAACAACACCGACCTGACCAACTACCGAAACCTGCTGTCTGGCGTGCTGTCTG<br>TGTCTATCTCTCAGGACGGCTCTCCCTTCGGCCTGCACTACATCATGTTCATGCCCGTGCTGCTGT<br>CTCAGGCCGACGAGAAGCAGCAAGAGAAGTGGCTGAAGCGAGCCATGAACTGCGAGATCATCGGC<br>TCTTACGCCCAGACCGAGCTTGGCCACGGCACCTTCATCCGAGGACTCGAGACTACCGCCACTTAC<br>GACCCCGCCACTCAAGAGTTCGTGCTGCACTCTCCCGCTCTGTCCTCTTACAAGTGGTGGCCCGGT<br>GGCCTGGGCAACACCGTGAACTACTGCATCGTGATCGCCCAGCTGTACTCTAAGGGCGTGTGCCA<br>CGGCATCCACTCGTTCATCGTGCAGGTCCGAGATGAGGACACCCACATGCCTCTGCCTGGCATCA<br>AGGTGGGCGAGATCGGCGTCAAGATGGGCCTGAACTCTGTGAACAACGGCTTCCTGGGCTTCGAG<br>AACGTGCGAATTCCCCGAGTGAACATGCTGATGAAGCACGCCAAGATTCTCGAGGACGGCACCTA<br>CGTGAAGTCTAAGAACAACAAGCTGATCTACGGCGCCATGGTGTTCGTGCGAGTGGTGATCGTGT<br>TCGACTCTGTCAACTACCTGGCCAAGGCCATCACTATCGGAGCCCGATACTCTCTGGTGCGACGA<br>CAGTCTCAGCTGAAGGCCGGCGAGCCCGAGCGACAGATCCTGGACTACGTGACCCAGCAGCACAA<br>GATTCTGCCCGCCATTGCCGGCTGCTACGCCATGAAGATGAACGCCTGGCGACTGTGGGACACCT<br>TTAACCTGATCAACGGCCAGCTGCACCAGGGCAACATGGAACGACTGGGCGAGCTGCACGCCCTG<br>GCCTGCTGCCTGAAGGCCATCTCTACCACCGACGCCGCTATGTTCACCTCTCTGTGCCGACTCGGC<br>TGTGGCGGCCACGGCTACATGACCTCTTCTAACCTGCCTCCTACCTACGCTCTGACCTCTGCCTCT<br>TGCACCTACGAGGGCGACAACACCGTTCTGCTGCTGCAGACCGCTCGATTCCTGCTCAAGACCTG<br>GCGACAGATTGACACCCATCCTCTGACTCGAACCGTGGCCTACCTCAAGACCGTGTCTGCTCCCGG<br>CTTCTCTGACCGATGGGAGTCCTCTGTCGAGGGCATCATTCGAGGCTTCCAGACCGTGGCTATGA<br>AGAAGATCTCTTCTTGCCTGGACATCATGACCTCCAAGGTGATGTCTGGCATGTCTCAAGAGGAC<br>GCCTGGAACGCCATCTCCATCCAGCTGGTGTCTGCCGCCGAGTCTCACTCTCGAGGCACCGTGATC

		TCTACCTTCTACGAGGACATGTCTAAGGCCATGCGATCTATGACCGCTCCTCTGGCTAAGGTGAT<br>GGGCCAGCTGGTCGAGCTGTACGCTGTGTACTGGACTCTCGAGCGACTGGGAGACATGCTGCAGT<br>ACACCTCTATCTCCCACACCGACGTGGTGGACCTGCGATCTTGGTACGAGGAACTCCTCCGAAAG<br>ATTCGACCCAACACCATCGGCCTGGTGGACGCCTTCGACATCATCGACGAGCTGCTCCAGTCTACC<br>CTGGGCGCCTACGACGGCCGAGTGTACGAGCGACTCATGGAAGAGGCCCTGAAGTCTCCCCTGAA<br>CGCTGAGCCCGTGAACCAGTCTTTCCACAAGTACCTGAAGCCTTTCATGCAGTCTAAGCTGTAA
Sequence No. 5	Peroxisomal oxidase 31760 from *Lobesia botrana*	ATGACTGAGGTGACCAAGGTGAACCCCGACCTCCAGCGAGAACGAGACAACTGTACATTCAACGT<br>TACCGAGCTGACTAACCTGATCGACGGCGGTGTGCAGAAGACCGAGGAGCGACGGAAGCGAGAGG<br>AGATGGTGCTGAAAGAAGGTATCCACCTGGACGAGGTTCCCTCAGAGTACCTGTCCCACAAGGAG<br>AAGTACGAGCTCGCTGTGAAGAAGGCATGCTACCTGTTCAAGATGATCCGACGACTGCAGGAGGA<br>AGAGAACACTGGAATGGAGAATTACCAGGAGGTGCTGGGCGGATCCCTGGGATCCGCTATCCTGC<br>GAGACGGCTCCCCTCTCACCCTGCATTACGTGATGTTCATCCCTACCATCATGGGCCAGGCTACGG<br>TTGAGCAGCAAGTGCTTTGGATCGGCCGAGCCTTCAATTGCGATATTATCGGAACTTACGCCCAG<br>ACTGAGCTTGGTCACGGAACCTTCATTCGAGGACTGGAAACCACCGCCACTTACGATCCCTCTAC<br>AAAGGAGTTCGTCCTGCACTCCCCCACTTTGACTTCCTACAAGTGGTGGCCCGGAGGCCTTGCTC<br>ACACGGCCAATTACTGTATCGTGGTGGCTCAACTTTACACACAAGGCAAGTGCCATGGCATTCAC<br>CCCTTTATCGTCCAGCTACGAGACGAAGAGACCCACATGCCCCTGCCCGGCATCAAGGTGGGCGA<br>AATCGGCGCTAAACTGGGAATGAACGGCACCAACAACGGCTTCCTTGGATTCGATAAGGTGCGAA<br>TCCCCCGAGAGCACATGCTGATGAAGAACGCCAAGGTTCTCGAGGACGGTACCTATGTGCGAGCC<br>CCCAGCAGTAAGCTGACATACGGTACTATGATGTTTGTGCGAGTTGTGCTGGTGAACGACGTGTG<br>CTCGTATATGGCAAAGGCCGTTACCATCGCTACTAGATACTCTGCTGTGCGACACCAGTCTCAGC<br>CTAAGCCCGACGAGCCCGAGCCCCAAATCCTGGAGTACGTGACTCAACAGCACAAGCTTGCCATC<br>GGCATTGCAACCGTGCACGCCTTTCGACTATCCGCCTCTTGGCTGTGGAACATGTACAACAACGT<br>GACAGCCGAGCTGGACGCCGGCGACTTAGAGCGACTTCCAGAACTGCACGCTCTGTCTTGCTGTC<br>TGAAGGCCGTTTCGACTGCTGATGCCTCTGAATGCGTCGAGCGATGCCGACTTGCTTGTGGAGGC<br>CACGGATACATGTTGTCCTCTAACTTCCCTCTGATGTACGGCATGGTGACCGCCGCTTGTACTTA<br>CGAAGGCGAGAACACCGTGATGCACCTGCAGACCGCCCGGTATCTGGTCAAGGCCTGGCAGCAGG<br>CCCAGGGTGGCAACCCCCTGACGCCTACTGTTGCCTACATCGGATCTATTGGCGCCGGACGACGA<br>AGCCCACCTTGGGATAACACCGTGGAGGGCATTATCCTGGGCTTCCAGCGCGTGGCTGCCGGTAA<br>GATTGCCCAGTGTGTTGCTAATATCGAAAAACGACAGCGAACCGGAATGTCTTACGAGGATGCTT<br>GGAACATGACCTCAGTGCAGCTTGTGTCTGCTAGCGAGGCCCACTGTCGAGCTTTCATCCTCCGG<br>ACCTACTTCGAGGAGACCGAAAACCAAATCGGTTCCGTCTCTCCCGCTCTGCGAGCAGTGCTGCT<br>GCAACTGGTCGATTTGTACGTTGTGTTTTGGGCTCTGCAGCGTGTCGGAGACCTTCTGAGATTCA<br>CCTCTATCTCTGAGCGAGACATCGAGCAGCTTCAGTCTTGGTACGAGGATCTTCTGATCAAGCTG<br>CGAGTCAACGCTGTCGGACTGGTCGACGCTTTCGACATCCGAGACGAGATCTTGAACTCTGCTCT<br>CGGAGCCTACGACGGGCGAGCTTACGAGCGGCTTATGGATGAAGCCCTTAAGTCTCCCCTGAACG<br>CTGAACCTGTGAACCAGTCTTTCCATAAGTATCTGAAGCCTTTCATGCAGGGTAAGCTGTAA
Sequence No. 6	Peroxisomal oxidase 49554 from *Lobesia botrana*	ATGGAGTCTAAGGACTTTGACCCTGTGTCCGATGCTGAGTTGAAAGACTACTTCCCCGACCTCCC<br>TTCTGGTCCTCTGGATAAGTTCCGAAAGAAGGCCACCTTCGACTGGAGACGAATGAAGCTAGTGT<br>ACGACTCTAAGCAGTCTATTGAGACCAAGGATAAGGTGTGGAAGTTCATGCTTGCTCACCCTCTG<br>TTCAAGCACTCTGTCGCCACCCCCACCCTGGATGAGCAGCGACAGATTGCTACCAAACGTATGTA<br>TCTGCTGCATAACGCTGACCTGGTCCCTCTGGAGGAAATCGCTATGCATCCCCGACTCTTTCAGTC<br>GTTGACTGAGGCCATTTTCATGTTCGACTCTTCGGTGGCCGTTAAGCTGTCTCTTACCTTCCGAA<br>TGTTCACCAACACAATTCGCGGATCGGGCAGACAGCATCACTACCACTTGATTGAGGACTGCGAC<br>AACGGCAAGATCGGCGGATGCTTCGCCCTGACTGAGATCGCCCACGGATCTAATGCTAAGGGCAT<br>GCGAACCACCGCAACCTACGACGTGGAGGGCCGATGTTTCGTCATGCACACCCCCGACTTTGAAG<br>CTGCCAAGTGTTGGGTTGGCTCCCTGGGTAAGTGTGCTACCCACGCCATTGTCTACGCCATGCTG<br>ATCTCTAAGGGCAAAAACCATGGTCTTCATTCCTTCGTGGTGCCCATCCGAGATCCTAAGACACT<br>CCGACCTTTTGCAGGCGTGACTGTCGGAGACATCGGCGAGAAGATCGGACTGAACGGAGTGGACA<br>ACGGTTTCCTGATGTTCAACAAGTACCGACTGCCCAAGGAGGCCGCTCTCGACAAGCTGGGTGGA<br>GTTGACGAAAACGGCGACTACAAGACTCCTTTCAGAGATCCTTCCAAGCGGTTCGGTGCTTCCCT<br>GGGCATTCTGTCTGGCGGCCGAGTGCACATTACCTCTATCTCTACCAACTACCTCCAGAAGGCTG<br>TGGTGATCGCTGTGCGATACTCTGCCGTGCGCCGACAGTTTGGCCCCGAGAACGCCGTCGAGGAG<br>ACTCCTGTCCTTGAGTACCAGCAACAGCAGATTCGACTTCTGCCCTACCTGGCCGCTACTTTCGCC<br>ATGCGAGTGTTCTGTAACTGGTTCGGCGAAGTGCACGTGCAGATGACCATCGATAACCTGGTTGG<br>TGCCGACGAGCAGGCTGCGGCCCGAGGTATCGAGATGCATGCCCTGTCTTCCGCCGTGAAGCCCG<br>TGTGCGGTTGGACCGCCCGAGACGGACTGCAGAACTGTAGAGAATGTTGCGGTGGTCATGGATAC<br>CTCCGAGCCTCCGCCATTGGCGACCTGCGAAACGACAACGACGCAAACTGTACCTACGAGGGCGA<br>GAACTCTATGCTCCTCCAGCAGACTTCTAACTGGCTGCTGGGCGTCTGGGCTAGACGACGACAGC<br>CCGGTTTCGCCGACACACCTTTCGGATCTATCCAATTCCTGGATGGCGCTCAGGCCCTGCTGGACG<br>AGTCTTGTCAGTGGAACTCCGCTGAAGAGATCGTGCAGCCTTCCAACGTCGTCCACATGTACAAG<br>TGGCTAACCACCTACATGCTCAAGCTCACCGCTGATAAGGTGTCCCAGCTTCGATCTCGAGGTTC<br>GTCTAACTACGAGGTCCGAAACGATTCGCAGTCCTACAACGCTGTGACTCTGTCTGTGGTGTATG<br>GAGAGCACTTTATTGTGAATCACTTCTACAAGACTGCACTGCAGTTCCAGGACGCCGCCTGTAGA<br>GCTGTCCTGCTGAAGCTGGTGGCCCTGTACGGAGCCTTCCTTCTCGAGAAGCACATGGCTACCCT<br>TTATATCGGAGGATTCTTTACCAAGCAGCAGGGACTGCTCCTGCGCGAAGGAGTTCTCTCTCTCT<br>GCAGCGCCCTGTCACCCCAGGCCGTGTCGCTGGCCGACACCCTCGCTCCCCCTGATTGGTGTCTGA<br>ACTCTGTGCTGGGATGCGCTGACGGCGAGGCGTACAAACATATCCAGGATTCTATTATGACTTAC<br>CCTGGCTCGATGACCCGGCCTGAGTGGTGGCGAGATGTTGCCCACTGGGAGACTTACGTGCCCGC<br>CAAGCTGTAA
Sequence No. 7	Peroxisomal oxidase from *Aspergillus nidulans*	ATGCCCAACCCTCCGCCTGCCTGGGTGCAAGCTCTGAAGCCCGCTTCGCCCCAGGGCACCGAGCTG<br>CTGACCCAAGAGCGAGCCCAGTCTAACATCGACGTGGACACCCTGGGCGACCTGCTGCACACCAA<br>GGAAGCCCTGAAGAAGCAGGACGAGATCCTGTCTGTGCTGAAGTCTGAGAAGGTGTTCGACAAG<br>TCTCGAAACCACGTGCTGGGCCGAACCGAGAAGATCCAGCTGGCCCTGGCTCGAGGCAAGCGACT

		GCAGCAGCTGAAGAAGGCCCACAACTGGTCTGACGAGGACGTCCACGTGGCCAACGACCTGGTGT<br>CTGAGCCCACTCCTTACGGCCTGCACGCCTCTATGTTTCTGGTGACCCTGCGAGAGCAGGGCACCC<br>CTGAGCAGCACAAGCTGTTCTACGAACGAGCCCGAAACTACGAGATCATCGGCTGCTACGCCCAG<br>ACCGAGCTTGGCCACGGCTCTAACGTGCGAGGACTCGAGACTACCGCCACTTGGGACCCCTCTGA<br>CCAGACCTTCATCATTCACTCTCCCACTCTGACCGCCTCTAAGTGGTGGATCGGCTCTCTGGGACG<br>AACCGCCAACCACGCCGTGGTGATGGCCCAGCTGTACATCGGCGGCAAGAACTACGGACCCCATC<br>CTTTCGTGGTGCAGATCCGAGACATGGAAACCCACCAGCCTCTCGAGAACGTGTACGTGGGCGAC<br>ATCGGCCCCAAGTTCGGCTACAACACCATGGACAACGGCTTTCTGCTGTTCAACAAGCTGAAGAT<br>TCCCCACGTGAACATGCTGGCCCGATTCGCCCAGGTGGACAAGGCCACCAACAAGTACATTCGAC<br>CCGCTTCTCCCTCTCTGATGTACGGCACCATGACCTGGGTGCGATCCAACATCGTGCTGCAGGCTG<br>GCGGCGTGCTCGCCCGAGGCGTGACCATTGCCGTGCGATACTGCGCCGTGCGACGACAGTTCCAG<br>GACCGAGATGCCAAGGCCAACGCCGAAGAGAACCAGGTGCTGAACTACAAGATGGTCCAGATTCG<br>ACTGCTGCCCCTGCTGGCCGCCATGTACGCCCTGCACTTCACCGGCCGAGGCATGATGCGACTGTA<br>CGAAGAAAACCAAGAACGAATGAAGGCTGCCGCTCAGGCCGACCAAGAGAAGCGAGGCGCTGGCC<br>CCGAGCAGCTGCGAGCCGGATCTGACCTGCTGGCTGACCTGCACGCTACCTCTTGCGGCCTGAAG<br>GCCCTGGCCTCTACCACCGCTGGCGAGGGCCTCGAGGTGTGCCGACGAGCCTGTGGCGGCCACGG<br>ATACTCTAACTACTCTGGCATCGGACCCTGGTACGCCGACTACCTGCCTACTCTGACCTGGGAGG<br>GCGACAACTACATGCTGACTCAGCAGGTTGCCCGATACCTGCTCAAGTCTGCCCGAGCCGTGCTG<br>GCCGGCAAGGGCACCGCCAACGACACCTCTCGAATCCTGCAGGCCTACCTCGCTCGACGAGACAA<br>GGGCGCCTCTTTCGACATCCTGGGCAACGACGCCGACATTGTGGCCGCCTTCGCCTGGCGAACCGC<br>TCACCTGACCTTCGAGACTCTGAAGTACCGAGATGTCGAGAAGCGATCTTGGAACTCTCTGCTGA<br>TCAACTTCTGGCGACTGTCTACCGCTCTGTCTCAGTACCTGGTGGTGAAGAACTTCTACGAGGCC<br>GTGAACTCTCCCGAGATCCGATCTTCTCTGGACAAGGACACTGCTTCTACCCTGCGATCTCTGTTC<br>CGACTGCACGCTCTGCACACCCTGGACCGAGAGGCCTCCGAGTTCTTCTCTTCTGCCGCCGTGACC<br>GTGCGACAGATCGGACTGACCCAGACCTCTGAGGTGCCCAAGCTGCTGGACGAGATTCGACCCCA<br>CGCCGTCCGACTGGTGGACTCTTGGAAGATCCCCGACTGGCAGCTGGACTCTGCCCTGGGCCGATC<br>TGACGGCGACGTGTACCCCGACCTGTTCAAGCGAGCCTCTATGCAGAACCCCGTGAACGACCTCG<br>TGTTCGACCCCTATCCTTGGAACGAGAACGTCCTGAAGAACGCCGGTGAGATCAAGTCTAAGCTG<br>TAG
Sequence No. 8	Peroxisomal oxidase from *Cucurbita maxima*	ATGGCCGCTGGCAAGGCCAAGGCTAAGATCGAGGTGGACATGGGATCTCTGTCTCTGTACATGCG<br>AGGCAAGCACCGAGAGATCCAAGAGCGAGTGTTCGAGTACTTCAACTCTCGACCCGAGCTGCAGA<br>CCCCTGTGGGCATCTCTATGGCCGACCACCGAGAGCTGTGCATGAAGCAGCTGGTCGGCCTGGTG<br>CGAGAGGCCGGCATTCGACCCTTCCGATTCGTGAACGAGGACCCCGCCAAGTACTTCGCCATCAT<br>GGAAGCCGTGGGCTCTGTGGACGTGTCTCTGGCCATCAAGATGGGCGTGCAGTTCTCTCTGTGGG<br>GCGGCTCTGTGATCAACCTGGGCACCAAGAAGCACCGGGACCGATTCTTCGACGGCATCGACAAC<br>GTGGACTACCCCGGCTGCTTCGCCATGACTGAGCTGCACCACGGCTCTAACGTGCAGGGCCTGCA<br>GACCACCGCCACTTTCGACCCCATCACCGACGAGTTCATCATCAACACCCCTAACGACGGCGCCAT<br>CAAGTGGTGGATCGGCAACGCCGCCGTCCACGGCAAGTTCGCCACCGTGTTCGCCAAGCTGGTGC<br>TGCCCACTCACGACTCTCGAAAGACCGCCGACATGGGAGTGCACGCCTTCATCGTGCCCATCCGAG<br>ATCTGAAGTCTCACAAGACCCTGCCTGGCATCGAGATCCACGACTGCGGCCACAAGGTGGGCCTG<br>AACGGCGTGGACAACGGCGCCCTGCGATTCCGATCTGTGCGAATTCCCCGAGACAACCTGCTGAA<br>CCGATTCGGCGAGGTGTCTCGAGATGGCAAGTACAAGTCCTCTCTGCCCTCTATCAACAAGCGAT<br>TCGCCGCCACTCTGGGCGAGCTGGTTGGCGGCCGAGTCGGACTGGCCTACTCTTCTGCCTCTGTGC<br>TGAAGATCGCCTCTACTATCGCCATCCGATACTCCCTGCTGCGACAGCAGTTCGGCCCTCCTAAGC<br>AGCCCGAGGTGTCCATCCTGGACTACCAGTCTCAGCAGCACAAGCTGATGCCCATGCTGGCCTCT<br>ACCTACGCCTTCCACTTCTCTACCATGCAGCTCGTCGAGAAGTACGCCCAGATGAAGAAGACCCA<br>CGACGAGGAACTGGTGGGCGACGTGCACGCCCTGTCTGCCGGCCTGAAGGCCTACGTGACCTCTT<br>ACACCGCCAAGTCTCTGTCTACCTGCCGAGAGGCCTGTGGCGGCCACGGATACGCCGTGGTCAAC<br>CGATTTGGCACCCTGCGAAACGACCACGACATCTTCCAGACCTTCGAGGGCGACAACACCGTGCT<br>GCTCCAGCAGGTCGCCGCCTACCTGCTCAAGCAGTACCAAGAGAAGTTCCAAGGCGGCACCCTGG<br>CCGTGACCTGGAACTACCTGCGAGAATCTATGAACACCTACCTCTCGCAGCCCAACCCTGTGACC<br>GCTCGATGGGAGTCTGCCGACCATCTGCGAGATCCCAAGTTTCAGCTGGACGCTTTCCAGTACCG<br>AACCTCTCGACTGCTGCAGTCTGTGGCCGTGCGACTGCGAAAGCACACCAAGAACCTGGGATCTT<br>TCGGCGCCTGGAACCGATGCCTGAACCATCTGCTGACCCTGGCTGAGTCTCACATCGAGTCTGTG<br>ATTCTGGCCCAGTTCATCGAGTCCGTGCAGAGATGTCCCAACGCTAACACCCAGGCTACCCTGAA<br>GCTGGTGTGCGACCTGTACGCTCTGGACCGAATCTGGAACGACATCGGCACCTACCGAAACGTCG<br>ACTACGTGGCTCCCAACAAGGCAAAGGCCATCCACAAGCTCACCGAGTACCTGTGCTTCCAGGTG<br>CGAAACATTGCCCAAGAGCTGGTGGACGCCTTCGACCTGCCTGACCACGTGACTCGAGCCCCTAT<br>TGCCATGAAGTCTAACGCCTACTCTCAGTACACCCAGTACATCGGCTTCGACCAGATTACCTCTG<br>TGGGATCTTCGTCTAAGCTGTAG
Sequence No. 9	Peroxisomal oxidase from *Homo sapiens*	ATGAACCCCGACCTGCGACGAGAGCGAGACTCTGCCTCTTTCAACCCCGAGCTGCTGACCCACATC<br>CTGGACGGCTCTCCCGAAAAGACCCGACGACGACGAGAAATCGAGAACATGATCCTGAACGACCC<br>CGACTTCCAGCACGAGGACCTGAACTTTCTGACCCGATCTCAGCGATACGAGGTGGCCGTGCGAA<br>AGTCTGCCATCATGGTGAAGAAGATGCGAGAGTTCGGAATCGCTGACCCCGACGAGATCATGTGG<br>TTCAAGAACTTCGTGCACCGAGGACGACCCGAGCCTCTGGACCTGCACCTGGGCATGTTTCTGCC<br>CACTCTGCTGCACCAGGCCACCGCCGAGCAGCAAGAGCGATTCTTCATGCCCGCCTGGAACCTCGA<br>GATCATCGGCACCTACGCTCAGACCGAGATGGGCCACGGCACCCACCTCCGAGGACTCGAGACTA<br>CCGCCACCTACGATCCCGAGACTCAAGAGTTCATCCTGAACTCTCCCACCGTGACCTCTATCAAGT<br>GGTGGCCCGGTGGACTGGGCAAGACCTCTAACCACGCCATCGTGCTGGCCCAGCTGATCACCAAG<br>GGCAAGTGCTACGGCCTGCACGCCTTCATCGTGCCCATCCGAGAGATCGGAACCCACAAGCCTCT<br>GCCTGGCATCACCGTGGGCGACATCGGCCCCAAGTTCGGCTACGACGAGATTGACAACGGCTACC<br>TGAAGATGGACAACCACCGAATTCCTCGAGAGAACATGCTGATGAAGTACGCCCAGGTGAAGCCC<br>GACGGAACCTACGTGAAGCCCCTGTCTAACAAGCTGACCTACGGAACCATGGTGTTCGTGCGATC<br>TTTCCTGGTCGGCGAGGCCGCTCGAGCCCTGTCCAAGGCCTGCACCATTGCCATCCGATACTCTGC

		CGTGCGACACCAGTCTGAGATCAAGCCCGGCGAGCCTGAGCCTCAGATCCTGGACTTTCAGACCC<br>AGCAGTACAAGCTGTTCCCTCTGCTGGCCACCGCTTACGCCTTCCAGTTCGTGGGCGCCTACATGA<br>AGGAAACCTACCATCGAATCAACGAAGGCATCGGCCAGGGCGACCTGTCTGAGCTGCCCGAACTG<br>CACGCCCTGACCGCCGGACTGAAGGCTTTCACCTCTTGGACCGCCAACACCGGCATCGAGGCCTGC<br>CGAATGGCCTGTGGCGGCCACGGCTACTCTCACTGCTCTGGACTGCCCAACATCTACGTGAACTTC<br>ACCCCTTCGTGTACCTTCGAGGGCGAGAACACCGTGATGATGCTGCAGACCGCTCGATTCCTCAT<br>GAAGTCTTACGACCAGGTGCACTCTGGCAAGCTGGTGTGCGGCATGGTGTCTTACCTGAACGATC<br>TGCCCTCTCAGCGAATTCAGCCTCAGCAGGTTGCCGTGTGGCCCACCATGGTCGACATCAACTCTC<br>CCGAGTCTCTGACCGAGGCCTACAAGCTGCGAGCTGCTCGACTGGTCGAGATCGCCGCCAAGAAC<br>CTGCAGAAGGAAGTCATCCACCGAAAGTCTAAGGAAGTGGCTTGGAACCTGACCTCTGTGGACCT<br>GGTGCGAGCTTCTGAGGCCCACTGCCACTACGTGGTGGTGAAGCTGTTCTCTGAGAAGCTGCTGA<br>AGATCCAGGACAAGGCCATCCAGGCCGTGCTGCGATCTCTGTGCCTGCTGTACTCTCTGTACGGC<br>ATCTCTCAGAACGCCGGCGACTTCCTGCAGGGCTCTATCATGACTGAGCCCCAGATTACCCAGGT<br>CAACCAGCGAGTGAAGGAACTGCTCACCCTGATCCGATCTGACGCCGTGGCTCTGGTGGACGCCT<br>TCGACTTTCAGGACGTGACCCTGGGCTCTGTGCTGGGCCGATACGACGGCAACGTGTACGAGAAC<br>CTGTTCGAGTGGGCCAAGAACTCGCCCCTGAACAAGGCCGAGGTGCACGAGTCTTACAAGCACCT<br>GAAGTCTCTGCAGTCTAAGCTGGACCAGATTACTTCTGTGGGATCTTCTTCGAAGCTGTAG
Sequence No. 10	Peroxisomal oxidase from *Paenarthrobacter ureafaciens*	ATGACCGAGGTGGTGGACCGAGCCTCTTCTCCCGCCTCTCCTGGCTCTACCACCGCCGCTGCCGAC<br>GGCGCCAAGGTGGCCGTCGAGCCTCGAGTGGACGTGGCCGCTCTGGGCGAGCAGCTCCTCGGCCG<br>ATGGGCCGACATCCGACTGCACGCCCGAGATCTGGCCGGACGAGAGGTGGTGCAGAAGGTCGAGG<br>GACTGACCCACACCGAGCACCGATCTCGAGTGTTCGGCCAGCTGAAGTACCTGGTGGACAACAAC<br>GCCGTGCACCGAGCTTTCCCTTCTCGACTCGGCGGATCTGACGACCACGGCGGCAACATTGCCGGC<br>TTCGAGGAACTGGTGACTGCTGACCCCTCGCTGCAGATCAAGGCCGGCGTCCAGTGGGGCCTGTT<br>CGGCTCTGCCGTGATGCACCTGGGCACCCGAGAGCACCACGACAAGTGGCTGCCCGGCATCATGT<br>CTCTCGAGATCCCCGGCTGCTTCGCCATGACCGAGACTGGCCACGGCTCTGACGTGGCCTCTATCG<br>CCACCACCGCCACCTACGACGAAGAGACTCAAGAGTTCGTGATCGACACACCCTTCCGAGCCGCCT<br>GGAAGGACTACATCGGCAACGCCGCCAACGACGGCCTGGCCGCCGTCGTGTTCGCTCAGCTGATC<br>ACCCGAAAGGTGAACCACGGCGTCCACGCCTTCTACGTGGACCTGCGAGATCCCGCCACCGGCGA<br>CTTTCTGCCCGGAATCGGCGGCGAGGACGACGGCATCAAGGGCGGCCTGAACGGCATCGACAACG<br>GACGACTGCACTTCACCAACGTGCGAATTCCCCGAACTAACCTGCTGAACCGATACGGCGACGTG<br>GCTGTGGACGGCACCTACTCTTCTACCATCGAGTCTCCCGGCCGACGATTCTTCACCATGCTGGGA<br>ACCCTGGTGCAGGGCCGAGTGTCTCTGGACGGCGCTGCCGTGGCCGCCTCTAAGGTGGCCCTGCA<br>GTCTGCCATCCACTACGCCGCCGAGCGACGACAGTTCAACGCCACCTCTCCTACCGAGGAAGAGG<br>TGCTGCTGGACTACCAGCGACATCAGCGACGACTGTTTACCCGACTGGCTACTACCTACGCTGCC<br>TCTTTCGCCCACGAACAGCTGCTGCAAAAGTTCGACGACGTGTTCTCTGGCGCCCACGACACCGA<br>CGCCGACCGACAGGACCTCGAGACTCTGGCTGCCGCTCTGAAGCCCCTGTCTACCTGGCACGCCCT<br>GGACACCCTGCAAGAGTGCCGAGAGGCCTGCGGCGGAGCCGGCTTCCTGATCGAGAACCGATTCG<br>CCTCTCTGCGAGCTGACCTGGACGTGTACGTGACCTTCGAGGGCGACAACACCGTGCTGCTGCAG<br>CTGGTGGCCAAGCGACTGCTGGCCGACTACGCCAAGGAATTCCGAGGCGCCAACTTCGGCGTGCT<br>GGCCCGATACGTCGTGGACCAGGCCGCTGGCGTGGCTCTGCACCGAACCGGCCTGCGACAGGTGG<br>CCCAGTTCGTGGCCGACTCCGGCTCTGTGCAGAAGTCTGCCCTGGCTCTGCGAGATGAGGAAGGC<br>CAGCGAACCCTGCTGACCGACCGAGTGCAGTCTATGGTGGCCGAGGTGGGCGCTGCCCTGAAGGG<br>CGCTGGCAAGCTGCCCCAGCACCAGGCTGCTGCCCTGTTCAACCAGCATCAGAACGAGCTGATCG<br>AGGCCGCTCAGGCCCACGCCGAGCTGCTCCAGTGGGAAGCCTTCACCGAGGCTCTGGCCAAGGTC<br>GACGACGCCGGCACCAAGGAAGTGCTGACCCGACTGCGGGACCTGTTCGGACTGTCTCTGATTGA<br>GAAGCACCTGTCTTGGTATCTGATGAACGGCCGACTGTCTATGCAGCGGGGACGAACCGTGGGCA<br>CCTACATCAACCGACTGCTCGTGAAGATTCGACCCCACGCTCTGGACCTGGTCGACGCCTTCGGCT<br>ACGGCGCTGAGCATCTGCGAGCCGCCATTGCCACCGGTGCCGAGGCCACTCGACAGGACGAGGCC<br>CGAACCTACTTCCGACAGCAGCGAGCCTCTGGATCTGCCCCTGCCGACGAAAAGACCCTGCTGGCC<br>ATTAAGGCCGGCAAGTCCCGAGATCAGATTACCTCTGTGGGATCTTCTTCGAAGCTGTAG
Sequence No. 11	Peroxisomal oxidase from *Rattus norvegicus*	ATGAACCCCGACCTGCGAAAGGAACGAGCCTCTGCCACTTTCAACCCCGAGCTGATCACCCACAT<br>CCTGGACGGCTCTCCCGAGAACACCCGACGACGACGAGAAATCGAGAACCTGATCCTGAACGACC<br>CCGACTTCCAGCACGAGGACTACAACTTTCTGACCCGATCTCAGCGATACGAGGTGGCCGTGAAG<br>AAGTCTGCCACCATGGTCAAGAAGATGCGAGAGTACGGCATCTCTGACCCCGAAGAGATCATGTG<br>GTTCAAGAACTCTGTGCACCGAGGACACCCTGAGCCTCTGGACCTGCACCTGGGCATGTTTCTGC<br>CCACTCTGCTGCACCAGGCTACCGCCGAGCAGCAAGAGCGATTCTTCATGCCCGCCTGGAACCTCG<br>AGATCACCGGCACCTACGCTCAGACCGAGATGGGCCACGGCACCCACCTCCGAGGACTCGAGACT<br>ACCGCCACTTACGACCCCAAGACTCAAGAGTTCATCCTGAACTCTCCCACCGTGACCTCTATCAAG<br>TGGTGGCCCGGTGGCCTGGGCAAGACCTCTAACCACGCCATCGTGCTGGCCCAGCTGATTACCCA<br>GGGCGAGTGCTACGGCCTGCACGCCTTCGTGGTGCCCATCCGAGAGATCGGAACCCACAAGCCAC<br>TGCCTGGCATCACCGTGGGCGACATCGGCCCCAAGTTCGGCTACGAGGAAATGGACAACGGCTAC<br>CTGAAGATGGACAACTACCGAATTCCTCGAGAGAACATGCTGATGAAGTACGCCCAGGTGAAGCC<br>CGACGGAACCTACGTGAAGCCCCTGTCTAACAAGCTGACCTACGGAACCATGGTGTTCGTGCGAT<br>CTTTCCTGGTGGGCAACGCCGCTCAGTCTCTGTCTAAGGCCTGCACCATTGCCATCCGATACTCTG<br>CCGTGCGACGACAGTCTGAGATCAAGCAGTCTGAGCCCGAGCCTCAGATCCTGGACTTTCAGACC<br>CAGCAGTACAAGCTGTTCCCTCTGCTGGCCACCGCCTACGCCTTCCACTTCGTGGGCCGATATATG<br>AAGGAAACCTACCTGCGAATCAACGAGTCTATCGGCCAGGGCGACCTGTCTGAGCTGCCCGAGCT<br>GCACGCCCTGACCGCCGGACTGAAGGCTTTCACCACCTGGACCGCCAACGCCGGCATCGAGGAAT<br>GCCGAATGGCCTGTGGCGGCCACGGCTACTCTCACTCCTCTGGCATCCCCAACATCTACGTGACCT<br>TCACTCCCGCCTGCACCTTCGAGGGTGAGAACACCGTGATGATGCTGCAGACCGCTCGATTCCTG<br>ATGAAGATCTACGACCAGGTGCGATCTGGCAAGCTGGTCGGCGGCATGGTGTCTTACCTGAACGA<br>TCTGCCCTCTCAGCGAATTCAGCCTCAGCAGGTTGCCGTGTGGCCCACTATGGTGGACATCAACT<br>CGCTCGAGGGCCTGACCGAGGCCTACAAGCTGCGAGCCGCTCGACTGGTCGAGATCGCCGCCAAG<br>AACCTGCAGACCCACGTGTCTCACCGAAAGTCTAAGGAAGTGGCTTGGAACCTGACCTCTGTGGA

		CCTGGTGCGAGCTTCTGAGGCCCACTGCCACTACGTGGTGGTGAAGGTGTTCTCTGACAAGCTGCCCAAGATCCAGGACAAGGCTGTCCAGGCCGTGCTGCGAAACCTGTGCCTGCTGTACTCTCTGTACGGAATCTCTCAGAAGGGCGGCGACTTCCTCGAGGGCTCTATCATCACCGGCGCTCAGCTGTCTCAGGTCAACGCTCGAATCCTCGAGCTGCTGACCCTGATTCGACCCAACGCCGTGGCTCTGGTGGACGCTTTCGACTTCAAGGACATGACCCTGGGCTCTGTGCTGGGACGATACGACGGCAACGTGTACGAGAACCTCTTCGAGTGGGCCAAGAAGTCTCCCCTGAACAAGACCGAGGTGCACGAGTCTTACCACAAGCACCTGAAGCCTCTGCAGTCTAAGCTGGACCAGATTACCTCCGTGGGATCTTCTTCGAAGCTGTAG
Sequence No. 12	Humanized renilla green fluorescent protein	ATGGTGAGCAAGCAGATCCTGAAGAACACCTGCCTGCAGGAGGTGATGAGCTACAAGGTGAACCTGGAGGGCATCGTGAACAACCACGTGTTCACCATGGAGGGCTGCGGCAAGGGCAACATCCTGTTCGGCAACCAGCTGGTGCAGATCCGCGTGACCAAGGGCGCCCCCCTGCCCTTCGCCTTCGACATCGTGAGCCCCGCCTTCCAGTACGGCAACCGCACCTTCACCAAGTACCCCAACGACATCAGCGACTACTTCATCCAGAGCTTCCCCGCCGGCTTCATGTACGAGCGCACCCTGCGCTACGAGGACGGCGGCCTGGTGGAGATCCGCAGCGACATCAACCTGATCGAGGACAAGTTCGTGTACCGCGTGGAGTACAAGGGCAGCAACTTCCCCGACGACGGCCCCGTGATGCAGAAGACCATCCTGGGCATCGAGCCCAGCTTCGAGGCCATGTACATGAACAACGGCGTGCTGGTGGGCGAGGTGATCCTGGTGTACAAGCTGAACAGCGGCAAGTACTACAGCTGCCACATGAAGACCCTGATGAAGAGCAAGGGCGTGGTGAAGGAGTTCCCCTCCTACCACTTCATCCAGCACCGCCTGGAGAAGACCTACGTGGAGGACGGCGGCTTCGTGGAGCAGCACGAGACCGCCATCGCCCAGATGACCAGCATCGGCAAGCCCCTGGGCAGCCTGCACGAGTGGGTGTAA
Sequence No. 13	mCherry	ATGGTGTCTAAGGGCGAAGAGGACAACATGGCCATCATCAAGGAATTCATGCGATTCAAGGTGCACATGGAAGGCTCTGTGAACGGCCACGAGTTCGAGATCGAAGGCGAAGGCGAGGGACGACCCTACGAGGGCACCCAGACCGCCAAGCTGAAGGTGACCAAGGGCGGACCCCTGCCTTTCGCCTGGGACATTCTGTCTCCCCAGTTCATGTACGGCTCTAAGGCCTACGTGAAGCACCCCGCCGACATTCCCGACTACCTGAAGCTGTCGTTCCCCGAGGGCTTCAAGTGGGAGCGAGTGATGAACTTCGAGGACGGCGGCGTGGTGACCGTGACTCAGGACTCTTCGCTGCAGGACGGCGAGTTCATCTACAAGGTGAAGCTGCGAGGCACCAACTTTCCCTCTGACGGCCCCGTGATGCAAAAGAAGACCATGGGCTGGGAAGCCTCTTCTGAGCGAATGTACCCCGAGGACGGTGCCCTGAAGGGCGAGATCAAGCAGCGACTGAAGCTCAAGGACGGTGGCCACTACGACGCCGAGGTCAAGACCACCTACAAGGCCAAGAAGCCCGTCCAGCTGCCTGGCGCCTACAACGTGAACATCAAGCTGGACATCACCTCTCACAACGAGGACTACACCATCGTCGAGCAGTACGAGCGAGCCGAGGGCCGACACTCTACCGGCGGCATGGACGAGCTGTACAAGTAG

Supplementary table S3. Biobricks used in this study

ID	Description	Forward primer	Reversed primer	Template DNA	Reference
BB8302	{-PrTEF1intron	PR-18928	PR-18975	Genomic DNA of ST4840	This study
BB2068	Har_FAR_Ylop->	PR-18066	PR-16595	Sequence No. 1	This study
BB2720	{-PrTEF1intron_USER_forfusion	PR-18930	PR-18214	Genomic DNA of ST4840	This study
BB8644	PrGPD_forfusion-}	PR-23004	PR-22213	Genomic DNA of ST4840	This study
BB8640	Dmd9{-PrTEF1intron_forfusion	PR-19018	PR-18930	Sequence No. 2	This study
BB2693	{-Lbo_PPTQ	PR-21723	PR-21724	Sequence No. 3	This study
BB1135	Vector backbone	See ref.	See ref.	See ref.	Holkenbrink 2018
BB8769	{-Ase_POX	PR-23435	PR-23436	Sequence No. 4	This study
BB2709	Lbo31670->	PR-21755	PR-21756	Sequence No. 5	This study
BB1558	PrExp-}	PR-15521	PR-15522	Genomic DNA of ST4840	This study
BB2710	Lbo49554->	PR-21757	PR-21758	Sequence No. 6	This study
BB1635	PrtRNA-Gly	See ref.	See ref.	See ref.	Holkenbrink 2018
BB1636	crRNA-TRPR	See ref.	See ref.	See ref.	Holkenbrink 2018
BB8516	{-Yli_POX2	PR-22827	PR-22828	Genomic DNA of ST4840	This study
BB8517	{-Yli_POX3	PR-22829	PR-22830	Genomic DNA of ST4840	This study
BB8519	{-Yli_POX5	PR-22833	PR-22834	Genomic DNA of ST4840	This study
BB8523	{-Ani_POX	PR-22841	PR-22842	Sequence No. 7	This study
BB8524	{-Cma_POX	PR-22843	PR-22844	Sequence No. 8	This study
BB8525	{-Hsa_POX	PR-22845	PR-22846	Sequence No. 9	This study
BB8526	{-Pur_POX	PR-22847	PR-22848	Sequence No. 10	This study
BB8527	{-Rno_POX	PR-22849	PR-22850	Sequence No. 11	This study
BB1688	->PrTEF1intron	See ref.	See ref.	See ref.	Petkevicius 2021

BB1740	Har_FAR_codoptYL	See ref.	See ref.	See ref.	Holkenbrink 2020
BB9309	PrTEF1intron_HarFAR_Per2	PR-10595	PR-24919	pBP8236	This study
BB8682	IntD_2_dwn	PR-23172	PR-23173	Genomic DNA of ST4840	This study
BB8681	IntD_2_up	PR-23171	PR-23170	Genomic DNA of ST4840	This study
BB2313	Fas2 (I1220F)	See ref.	See ref.	See ref.	Petkevicius 2021
BB8679	IntE_7_up	PR-23167	PR-23166	Genomic DNA of ST4840	This study
BB8680	IntE_7_dwn	PR-23168	PR-23169	Genomic DNA of ST4840	This study
BB1631	TPex20-TLip2	See ref.	See ref.	See ref.	Holkenbrink 2018
BB8386	IntF_5_Up	PR-22532	PR-22533	Genomic DNA of ST4840	This study
BB8387	IntF_5_Down	PR-22534	PR-22535	Genomic DNA of ST4840	This study
BB10144	PrTEF1intronHarFARforhrGFP	PR-10595	PR-26919	pBP8236	This study
BB10145	hrGFPforHarFAR	PR-26920	PR-15506	Sequence No. 12	This study
BB10146	hrGFPforHarFAR_Per2	PR-26920	PR-26921	Sequence No. 12	This study
BB2093	PrTEF1intron_USER-}	See ref.	See ref.	See ref.	Petkevicius 2021
BB10154	YliPOX3formCherry	PR-24936	PR-26932	Genomic DNA of ST4840	This study
BB10155	YliALE1formCherry	PR-26926	PR-26933	Genomic DNA of ST4840	This study
BB10156	mCherryforYliPOX3	PR-26934	PR-26936	Sequence No. 13	This study
BB10157	mCherryforYliALE1	PR-26935	PR-26936	Sequence No. 13	This study

Supplementary table S4. Plasmids used in this study

ID	Description	Parent plasmid	Biobricks/primers	Reference
pCfB6630	pNat-YLgRNA3_IntC_3	See ref.	See ref.	Holkenbrink 2018
pBP8754	pIntF_3-Ase_POX_GeneArt{-PrEXP	pBP8009	BB2721, BB8769	This study
pBP8627	pIntD_2-Dmd9{-PrTEF1intron-PrGPD-}HarFAR	pBP8620	BB8640, BB2720, BB8644, BB2068	This study
pBP8400	pIntC_3-TPex20-PrEXP-}Lbo31670-TLip2	pCfB6371	BB2709, BB1558	This study
pBP8401	pIntC_3-TPex20-PrEXP-}Lbo49554-TLip2	pCfB6371	BB2710, BB1558	This study
pBP8802	pIntD_2-Lbo_PPTQ{-PrTEF1intron-PrGPD-}HarFAR	pBP8620	BB2693, BB2720, BB8644, BB2068	This study
pCfB7088	pNat-YLgRNA1_Fas2 (AA1220)	See ref.	See ref.	Holkenbrink 2020
pBP8900	pHph_YLgRNA5_IntE_4	pCfB3431	BB1635, BB1636, PR-23285, PR-23286	This study
pBP8340	pIntC_3-TPex20-Yli_POX2{-PrTEF1-TLip2	pCfB6371	BB8516, BB8302	This study
pBP8341	pIntC_3-TPex20-Yli_POX3{-PrTEF1-TLip2	pCfB6371	BB8517, BB8302	This study
pBP8343	pIntC_3-TPex20-Yli_POX5{-PrTEF1-TLip2	pCfB6371	BB8519, BB8302	This study
pBP8347	pIntC_3-TPex20-Ani_POX{-PrTEF1-TLip2	pCfB6371	BB8523, BB8302	This study
pBP8348	pIntC_3-TPex20-Cma_POX{-PrTEF1-TLip2	pCfB6371	BB8524, BB8302	This study
pBP8349	pIntC_3-TPex20-Hsa_POX{-PrTEF1-TLip2	pCfB6371	BB8525, BB8302	This study
pBP8350	pIntC_3-TPex20-Pur_POX{-PrTEF1-TLip2	pCfB6371	BB8526, BB8302	This study
pBP8351	pIntC_3-TPex20-Rno_POX{-PrTEF1-TLip2	pCfB6371	BB8527, BB8302	This study
pBP8003	pNat-YLgRNA4_IntF_3	pCfB3405	BB1635, BB1636, PR-22039, PR-22040	This study
pBP8623	pNat_YLgRNA1_IntD_2	pCfB3405	BB8736	This study
pBP8576	pHph_YLgRNA1_IntD_2	pCfB3431	BB1635, BB1636, PR-23192, PR-23193	This study

pBP8032	pHph-YLgRNA3_IntC_3	pCfB3431	BB1635, BB1636, PR-18239, PR-18240	This study
pBP8236	pIntE_4-PrTEF1intron->HarFAR	pCf6679	BB1688, BB1740	This study
pBP9438	pIntE_4_PrTEF1intron_HarFAR_Per2	pCf6679	BB9309	This study
pBP8009	pIntF_3-TPex20-TLip2		BB1135, BB1631, BB8031, BB1480	This study
pBP6371	pIntC_3-TPex20-TLip2	See ref.	See ref.	Holkenbrink 2018
pBP3405	pORI1001-Nat-CEN1-USER	See ref.	See ref.	Holkenbrink 2018
pBP3431	pORI1001-Hphsyn-CEN1-USER	See ref.	See ref.	Holkenbrink 2020
pBP6679	pIntE_4-TPex20-TLip2	See ref.	See ref.	Holkenbrink 2018
pBP8620	pIntD_2-TPex20-TLip2		BB1135, BB8682, BB8681	This study
pBP8575	pNat_YLgRNA1_IntE_7		BB1635, BB1636, PR-23190, PR-23191	This study
pBP8645	pHph_YLgRNA4_IntF_5		BB1635, BB1636, PR-23127, PR-23128	This study
pBP8662	pIntE_7-TPex20-TLip2		BB1135 ,BB8679, BB8680, BB1631	This study
pBP8263	IntF_5_Up_TPex20-USER-TLip2_IntF_5_Down		BB1135 ,BB8386, BB1631, BB8387	This study
pBP10672	pIntE_7-TPex20-PrTEF1intron_HarFAR_hrGFP-TLip2	pBP8662	BB10144, BB10145	This study
pBP10669	pIntE_7-TPex20- PrTEF1intron _HarFAR_hrGFP_Per2-TLip2	pBP8662	BB10144, BB10146	This study
pBP10676	IntF_5_Up_TPex20- PrTEF1intron _YliPOX3_mCherry-TLip2_IntF_5_Down	pBP8263	BB2093, BB10154, BB10156	This study
pBP10677	IntF_5_Up_TPex20- PrTEF1intron _YliALE1_mCherry-TLip2_IntF_5_Down	pBP8263	BB2093, BB10155, BB10157	This study

Supplementary table S5. Strains used in this study

ID	Relevant features	Parent strain	Added elements	Reference/source
ST4840	Wild-type *Yarrowia lipolytica*			Agricultural Research Service (NRRL, USA)
ST6629	See ref.	See ref.	See ref.	Holkenbrink 2020
ST8524	See ref.	See ref.	See ref.	Petkevicius 2021
ST9138	ΔPOX1-6	See ref.	See ref.	Patent application WO/2020/169389
ST9199	ΔPOX1-6, IntC_3-Yli_POX2{-TEF1	ST9138	pCfB6630, pBP8340	This study
ST9200	ΔPOX1-6, IntC_3-Yli_POX3{- TEF1	ST9138	pCfB6630, pBP8341	This study
ST9202	ΔPOX1-6, IntC_3-Yli_POX5{- TEF1	ST9138	pCfB6630, pBP8343	This study
ST9206	ΔPOX1-6, IntC_3-Ani_POX{- TEF1	ST9138	pCfB6630, pBP8347	This study
ST9207	ΔPOX1-6, IntC_3-Cma_POX{- TEF1	ST9138	pCfB6630, pBP8348	This study
ST9208	ΔPOX1-6, IntC_3-Hsa_POX{- TEF1	ST9138	pCfB6630, pBP8349	This study
ST9209	ΔPOX1-6, IntC_3-Pur_POX{- TEF1	ST9138	pCfB6630, pBP8350	This study
ST9210	ΔPOX1-6, IntC_3-Rno_POX{- TEF1	ST9138	pCfB6630, pBP8351	This study
ST9284	ΔPOX1-6, IntF_3-Ase_POX{-PrEXP	ST9138	pBP8754, pBP8003	This study
ST9294	ΔPOX1-6, IntD_2-Dmd9{-PrTEF1intron-PrGPD-}HarFAR	ST9138	pBP8627, pBP8623	This study
ST9295	ΔPOX1-6, IntC_3-Yli_POX2{-PrEXP, IntD_2-Dmd9{-PrTEF1intron-PrGPD-}HarFAR	ST9199	pBP8627, pBP8576	This study
ST9296	ΔPOX1-6, IntC_3-Yli_POX3{-PrEXP, IntD_2-Dmd9{-PrTEF1intron -PrGPD-}HarFAR	ST9200	pBP8627, pBP8576	This study
ST9297	ΔPOX1-6, , IntC_3-Yli_POX5{-PrEXP IntD_2-Dmd9{- PrTEF1intron-PrGPD-}HarFAR	ST9202	pBP8627, pBP8576	This study
ST9298	ΔPOX1-6, IntC_3-Ani_POX{-PrEXP, IntD_2-Dmd9{-PrTEF1intron-PrGPD-}HarFAR	ST9206	pBP8627, pBP8576	This study
ST9299	ΔPOX1-6, IntC_3-Cma_POX{-PrEXP, IntD_2-Dmd9{- PrTEF1intron-PrGPD-}HarFAR	ST9207	pBP8627, pBP8576	This study
ST9300	ΔPOX1-6, IntC_3-Hsa_POX{-PrEXP, IntD_2-Dmd9{- PrTEF1intron-PrGPD-}HarFAR	ST9208	pBP8627, pBP8576	This study

ST9301	ΔPOX1-6, IntC_3-Pur_POX{-PrEXP, IntD_2-Dmd9{- PrTEF1intron-PrGPD-}HarFAR	ST9209	pBP8627, pBP8576	This study
ST9302	ΔPOX1-6, IntC_3-Rno_POX{-PrEXP, IntD_2-Dmd9{- PrTEF1intron-PrGPD-}HarFAR	ST9210	pBP8627, pBP8576	This study
ST9329	ΔPOX1-6, IntF_3-Ase_POX{-PrEXP, IntD_2-Dmd9{- PrTEF1intron-PrGPD-}HarFAR	ST9284	pBP8627, pBP8576	This study
ST9347	ΔPOX1-6, IntD_2-Dmd9{-PrTEF1intron-PrGPD-}HarFAR, IntC_3-PrEXP-}Lbo_31670	ST9294	pBP8400, pBP8032	This study
ST9348	ΔPOX1-6, IntD_2-Dmd9{-PrTEF1intron-PrGPD-}HarFAR, IntC_3-PrEXP-}Lbo_49554	ST9294	pBP8401, pBP8032	This study
ST9314	ΔPOX1-6, ΔPOX1-6, IntD_2-Lbo_PPTQ{- PrTEF1intron -PrGPD-}HarFAR	ST9138	pBP8802, pBP8623	This study
ST9315	ΔPOX1-6, IntC_3-Yli_POX2{-PrEXP, IntD_2-Lbo_PPTQ{- PrTEF1intron-PrGPD-}HarFAR	ST9199	pBP8802, pBP8576	This study
ST9316	ΔPOX1-6, IntC_3-Yli_POX3{-PrEXP, IntD_2-Lbo_PPTQ{- PrTEF1intron-PrGPD-}HarFAR	ST9200	pBP8802, pBP8576	This study
ST9317	ΔPOX1-6, IntC_3-Yli_POX5{-PrEXP, IntD_2-Lbo_PPTQ{- PrTEF1intron-PrGPD-}HarFAR	ST9202	pBP8802, pBP8576	This study
ST9318	ΔPOX1-6, IntC_3-Ani_POX{-PrEXP, IntD_2-Lbo_PPTQ{- PrTEF1intron-PrGPD-}HarFAR	ST9206	pBP8802, pBP8576	This study
ST9319	ΔPOX1-6, IntC_3-Cma_POX{-PrEXP, IntD_2-Lbo_PPTQ{-PrTEF1intron-PrGPD-}HarFAR	ST9207	pBP8802, pBP8576	This study
ST9320	ΔPOX1-6, IntC_3-Hsa_POX{-PrEXP, IntD_2-Lbo_PPTQ{- PrTEF1intron-PrGPD-}HarFAR	ST9208	pBP8802, pBP8576	This study
ST9321	ΔPOX1-6, IntC_3-Pur_POX{-PrEXP, IntD_2-Lbo_PPTQ{- PrTEF1intron-PrGPD-}HarFAR	ST9209	pBP8802, pBP8576	This study
ST9322	ΔPOX1-6, IntC_3-Rno_POX{-PrEXP, IntD_2-Lbo_PPTQ{- PrTEF1intron-PrGPD-}HarFAR	ST9210	pBP8802, pBP8576	This study
ST9330	ΔPOX1-6, IntF_3-Ase_POX{-PrEXP, IntD_2-Lbo_PPTQ{- PrTEF1intron-PrGPD-}HarFAR	ST9284	pBP8802, pBP8576	This study
ST9350	ΔPOX1-6, IntD_2-Lbo_PPTQ{-PrTEF1intron -PrGPD-}HarFAR, IntC_3-PrEXP-}Lbo_31670	ST9314	pBP8400, pBP8032	This study
ST9351	ΔPOX1-6, IntD_2-Lbo_PPTQ{-PrTEF1intron -PrGPD-}HarFAR, IntC_3-PrEXP-}Lbo_49554	ST9314	pBP8401, pBP8032	This study
ST10313	ΔPOX1-6, IntD_2-Dmd9{-PrTEF1intron -PrGPD-}HarFAR, IntC_3-PrEXP-}Lbo_31670, FAS2 (I1220F)	ST9347	pCfB7088, BB8908	This study
ST10383	ΔPOX1-6, IntD_2-Dmd9{-PrTEF1intron -PrGPD-}HarFAR, IntC_3-PrEXP-}Lbo_31670, FAS2 (I1220F), IntE_4- PrTEF1intron ->HarFAR	STST10313	pBP8900, pBP8236	This study
ST10384	ΔPOX1-6, IntD_2-Dmd9{-PrTEF1intron -PrGPD-}HarFAR, IntC_3-PrEXP-}Lbo_31670, FAS2 (I1220F), IntE_4_ PrTEF1intron _HarFAR_Per2	STST10313	pBP8900, pBP9438	This study
ST10314	ΔPOX1-6, IntD_2-Lbo_PPTQ{-PrTEF1intron -PrGPD-}HarFAR, IntC_3-PrEXP-}Lbo_31670, FAS2 (I1220F)	ST9350	pCfB7088, BB8908	This study
ST10387	ΔPOX1-6, IntD_2-Lbo_PPTQ{-PrTEF1intron -PrGPD-}HarFAR, IntC_3-PrEXP-}Lbo_31670, FAS2	ST10314	pBP8900, pBP8236	This study

	(I1220F), IntE_4- PrTEF1intron ->HarFAR			
ST10388	ΔPOX1-6, IntD_2-Lbo_PPTQ{-PrTEF1intron -PrGPD-}HarFAR, IntC_3-PrEXP-}Lbo_31670, FAS2 (I1220F), IntE_4- PrTEF1intron ->HarFAR_Per2	ST10314	pBP8900, pBP9438	This study
ST12413	ΔPOX1-6, IntE_7- PrTEF1intron _HarFAR_hrGFP	ST9138	pBP8575, pBP10672	This study
ST12410	ΔPOX1-6, IntE_7- PrTEF1intron _HarFAR_hrGFP_Per2	ST9138	pBP8575, pBP10669	This study
ST12433	ΔPOX1-6, IntE_7- PrTEF1intron _HarFAR_hrGFP, IntF_5-PrTEF1intron _YliPOX3_mCherry-	ST12413	pBP8645, pBP10676	This study
ST12434	ΔPOX1-6, IntE_7- PrTEF1intron _HarFAR_hrGFP, IntF_5-PrTEF1intron _YliALE1_mCherry-	ST12413	pBP8645, pBP10677	This study
ST12424	ΔPOX1-6, IntE_7- PrTEF1intron _HarFAR_hrGFP_Per2, IntF_5-PrTEF1intron _YliPOX3_mCherry-TLip2_IntF_5_Down	ST12410	pBP8645, pBP10676	This study
ST12425	ΔPOX1-6, IntE_7- PrTEF1intron _HarFAR_hrGFP_Per2, IntF_5-PrTEF1intron _YliALE1_mCherry-TLip2_IntF_5_Down	ST12410	pBP8645, pBP10677	This study

Supplementary materials and methods relevant for generation of data presented in supplementary table S6 and supplementary figure S7.

Y. lipolytica strains were inoculated into 37.5 mL of YPG media in 250 mL shake flasks at OD_{600}=0.2 and cultivated for 22 h at 28°C, shaken at 300 rpm at 5 cm orbit cast. Cell growth over time was monitored by measuring optical density using GENESYS™ 10S UV-Vis Spectrophotometer (Thermo Scientific). After 22 h of growth, cultivation broth was transferred into 50 mL falcon tube, centrifuged for 5 min at room temperature at 3000x*g*. The supernatant was discarded, and the cells resuspended in 20 mL production medium. Resuspended cells were transferred back to shake flasks and 0.4% (v/v) of 14:Me supplied. For cell dry weight and glycerol measurements, 1 mL cultivation broth was taken and centrifuged for 5 min at room temperature at 12500x*g*. Supernatant was used for glycerol measurements. Cell pellet was resuspended in 1 mL 70% ethanol and centrifuged for 5 min at room temperature at 12500x*g*. Liquid was removed and pellet was kept for 72 h in the 70°C degrees oven before weight measurements. Glycerol was measured using Randox GY105 glycerol assay. Quantification of 14:Me and Z7-12:OH was performed using GC-FID under the same settings as for data presented in Figures S1 and S2. Quantification of Z9-12:OH was performed under the same settings as for data presented in Figure 2.

Supplementary table S6. Titers and specific yields (g of product/g of dry weight) of Z7-12:OH, Z9-12:OH and Z7-14:OH at the end of shake flask cultivations (52h after media exchange).

Strain \ Product	Z7-12:OH	Z9-12:OH	Z7-14:OH
ST10384	1.13±0.05 mg/L<br>Y_{xp}=4.8x10^{-5}±4.4x10^{-6}	0 mg/L<br>Y_{xp}=0	0.15±0.04 mg/L<br>Y_{xp}=6.4x10^{-6}±2.1x10^{-6}
ST10388	0 mg/L<br>Y_{xp}=0	0.33±0.07 mg/L<br>Y_{xp}=1.1x10^{-5}±1.9x10^{-6}	0.13±0.06 mg/L<br>Y_{xp}=4.3x10^{-6}±2.2x10^{-6}

Supplementary references:

Holkenbrink C, Dam MI, Kildegaard KR *et al*. EasyCloneYALI: CRISPR/Cas9-Based Synthetic Toolbox for Engineering of the Yeast *Yarrowia lipolytica*. *Biotechnol J* 2018; 13:1–8.

Holkenbrink C, Ding BJ, Wang HL, *et al*. Production of moth sex pheromones for pest control by yeast fermentation. *Metab Eng* 2020, DOI:10.1101/2020.07.15.205047.

Petkevicius K, Koutsoumpeli E, Betsi PC *et al*. Biotechnological production of the European corn borer sex pheromone in the yeast *Yarrowia lipolytica*. *Biotechnol J* 2021, DOI:10.1002/biot.202100004

Chromatogram number	Strain ID	FAD	FAR	Peroxisomal oxidase
1	ST9294	Dmd9	HarFAR	-
2	ST9295			YliPOX2
3	ST9296			YliPOX3
4	ST9297			YliPOX5
5	ST9298			AniPOX
6	ST9299			CmaPOX
7	ST9300			HsaPOX
8	ST9301			PurPOX
9	ST9302			RnoPOX
10	ST9329			AsePOX
11	ST9347			Lbo_31670
12	ST9348			Lbo_49554

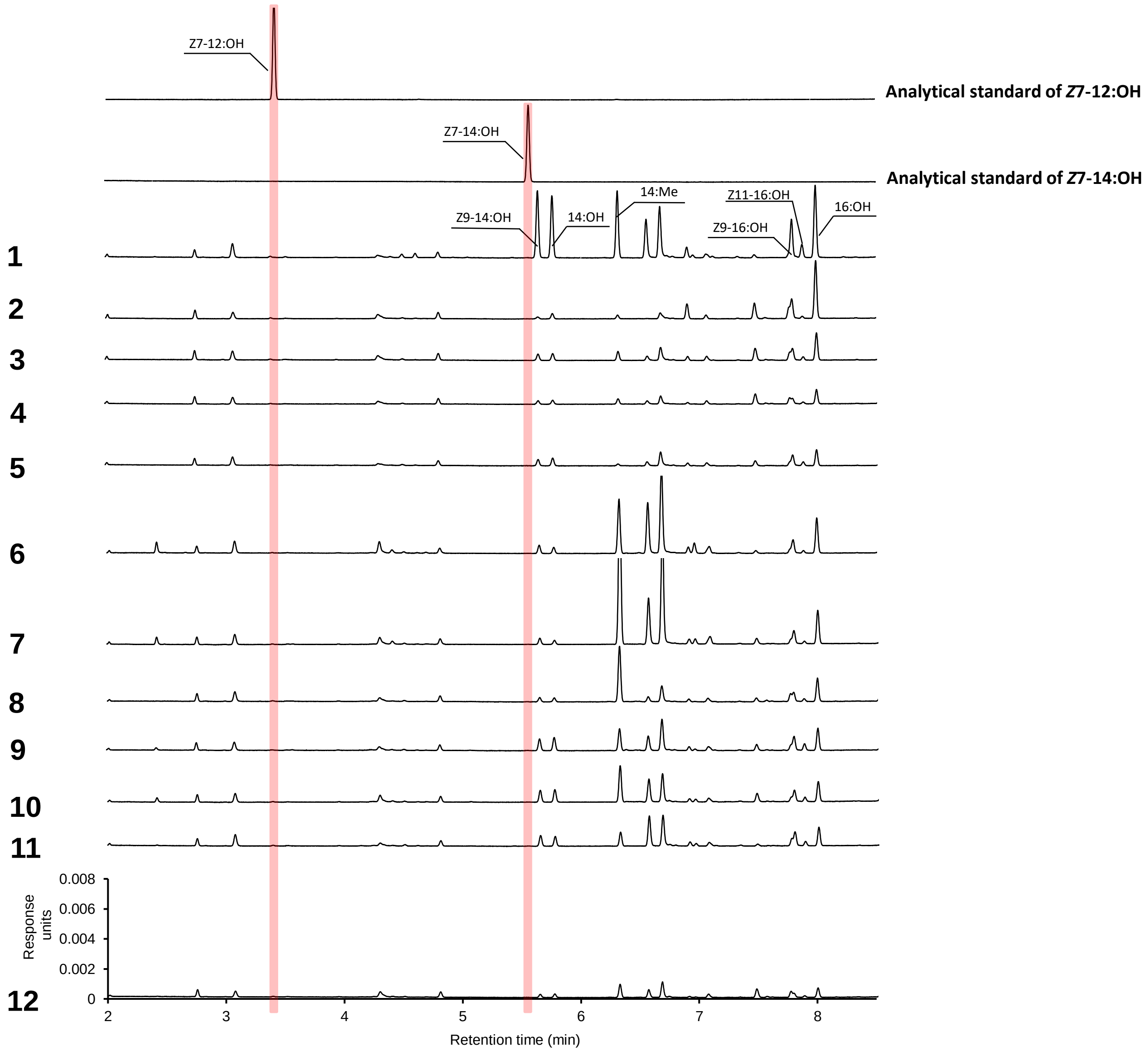

Figure S1. Fatty alcohol profiles obtained from the *Y. lipolytica* strains containing Dmd9, HarFAR and different POXes. Cultivation media was supplemented with 0.24% (v/v) of 14:Me.

Chromatogram number	Strain ID	FAD	FAR	Peroxisomal oxidase
1	ST9314	Lbo_PPTQ	HarFAR	-
2	ST9315			YliPOX2
3	ST9316			YliPOX3
4	ST9317			YliPOX5
5	ST9318			AniPOX
6	ST9319			CmaPOX
7	ST9320			HsaPOX
8	ST9321			PurPOX
9	ST9322			RnoPOX
10	ST9330			AsePOX
11	ST9350			Lbo_31670
12	ST9351			Lbo_49554

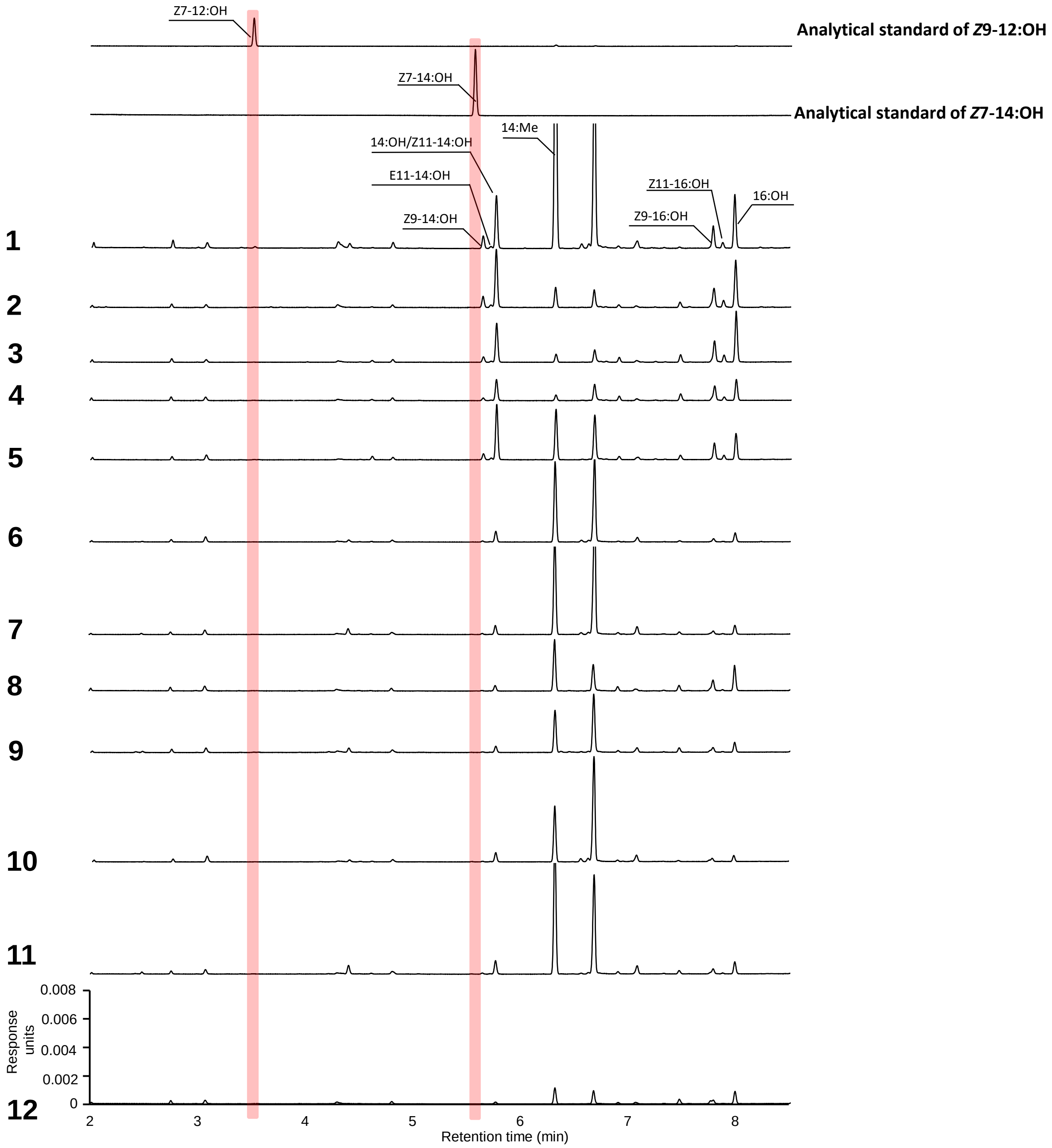

Figure S2. Fatty alcohol profiles obtained from the *Y. lipolytica* strains containing Lbo_PPTQ, HarFAR and different POXes. Cultivation media was supplemented with 0.24% (v/v) of 14:Me.

% of Z7-12:Me in total FAMEs	0.004	0.032	0	0	0	0	0.185	0.093	0.195	0.046	0.036	0.370	0.0.03
% of Z9-12:Me in total FAMEs	0	0	0	0	0	0	0	0	0	0	0	0	0
% of Z7-14:Me in total FAMEs	0	0	0	0	0	0	0.023	0.012	0.014	0.021	0	0.037	0.032

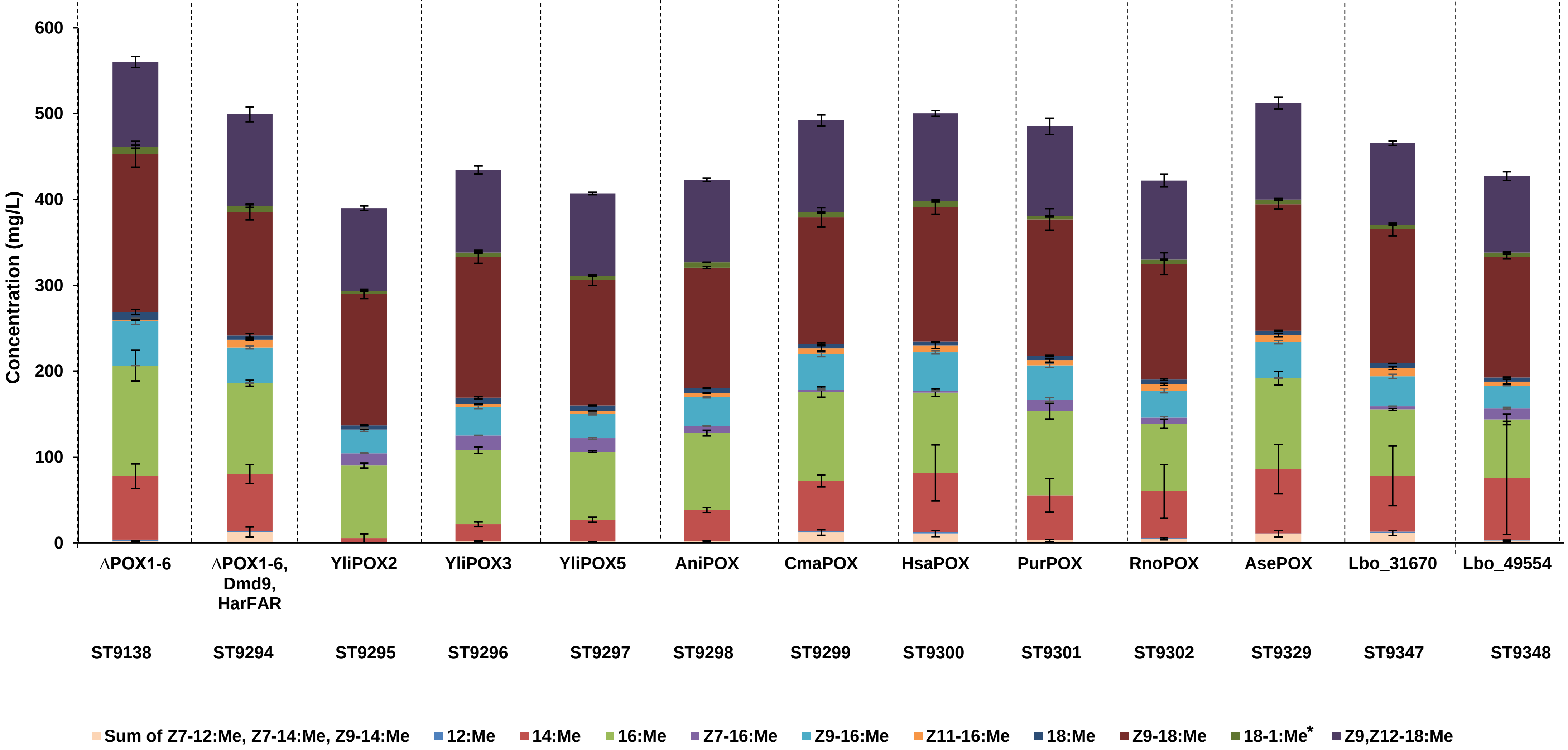

Figure S3. Fatty acid profiles in the form of methyl esters obtained from the *Y. lipolytica* strains containing Dmd9, HarFAR and different POXes. Cultivation media was supplemented with 0.24% (v/v) of 14:Me. *: Position of double bond remains to be identified. Error bars represent standard deviations from three technical replicates

% of Z7-12:Me in total FAMEs	0	0	0	0	0	0	0	0	0	0	0.014	0
% of Z9-12:Me in total FAMEs	0.037	0	0	0	0	0.037	0.090	0.010	0.029	0.081	0.126	0.011
% of Z7-14:Me in total FAMEs	0	0	0	0	0	0.006	0.006	0.005	0.015	0	0.010	0.014

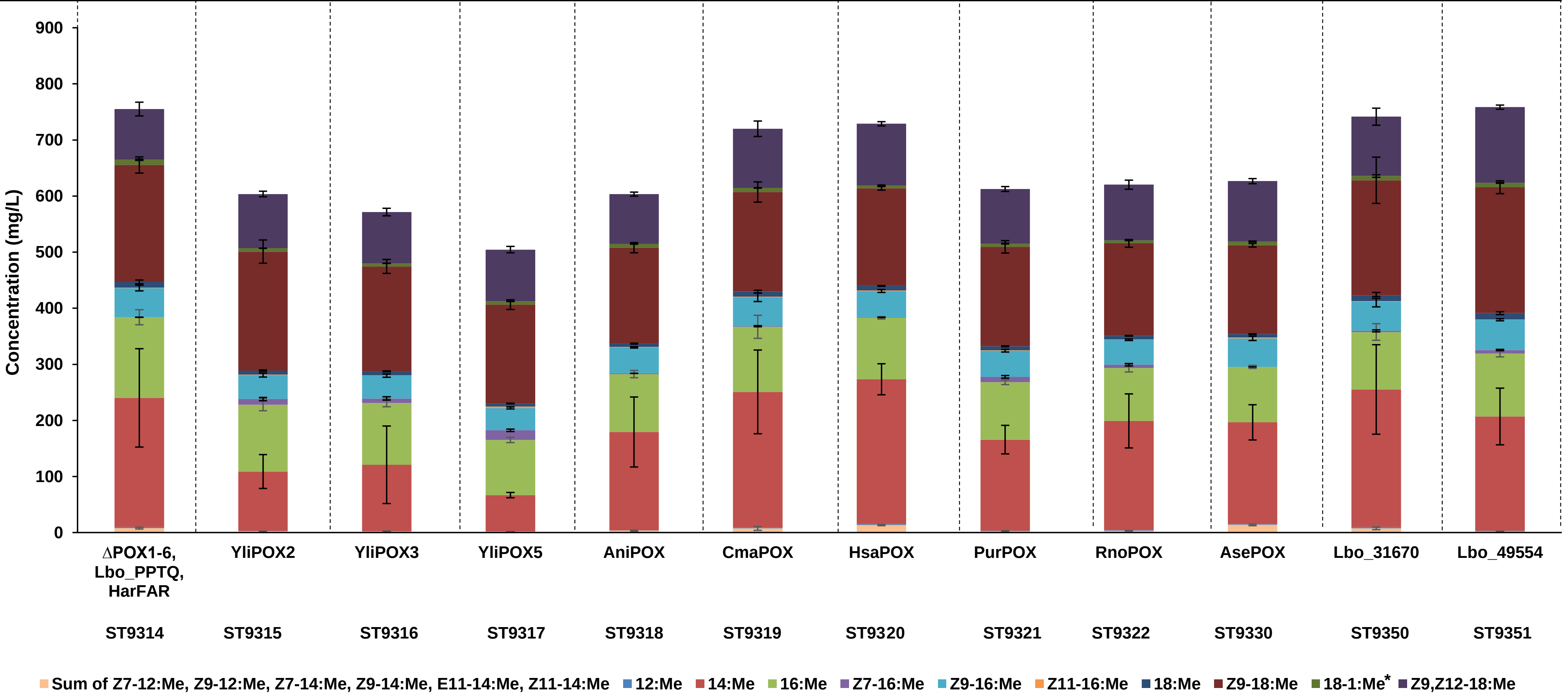

Figure S4. Fatty acid profiles in the form of methyl esters obtained from the *Y. lipolytica* strains containing Lbo_PPTQ, HarFAR and different POXes. Cultivation media was supplemented with 0.24% (v/v) of 14:Me. *: Position of double bond remains to be identified. Error bars represent standard deviations from three technical replicates

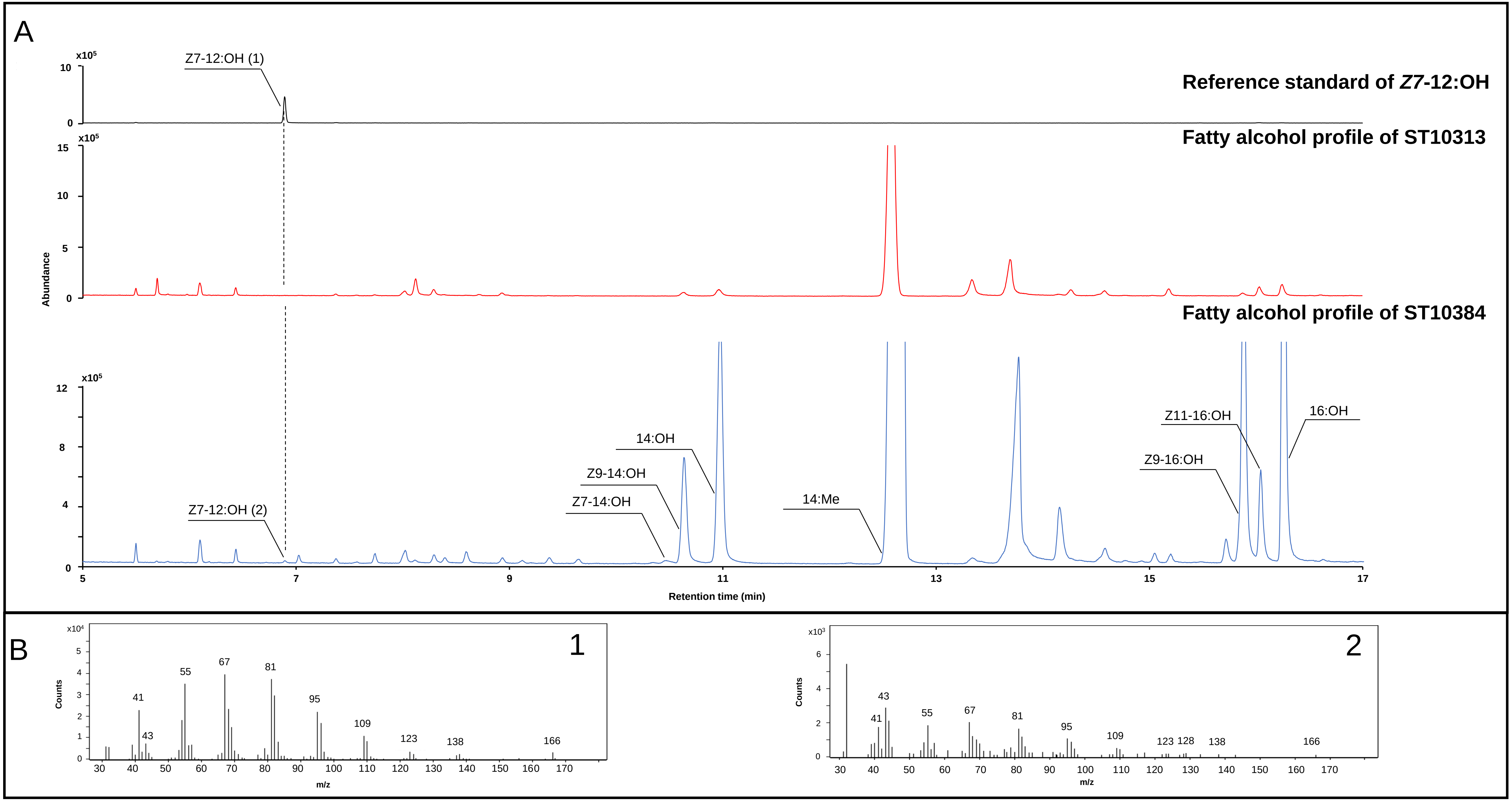

Figure S5.A. GC-MS chromatograms of Z7-12:OH reference standard and fatty alcohol profiles of ST10313 (red) and ST10384 (blue). B. Mass spectra of Z7-12:OH reference standard (1) and biologically produced Z7-12:OH (2). Cultivation media was supplemented with 0.4% (v/v) of 14:Me.

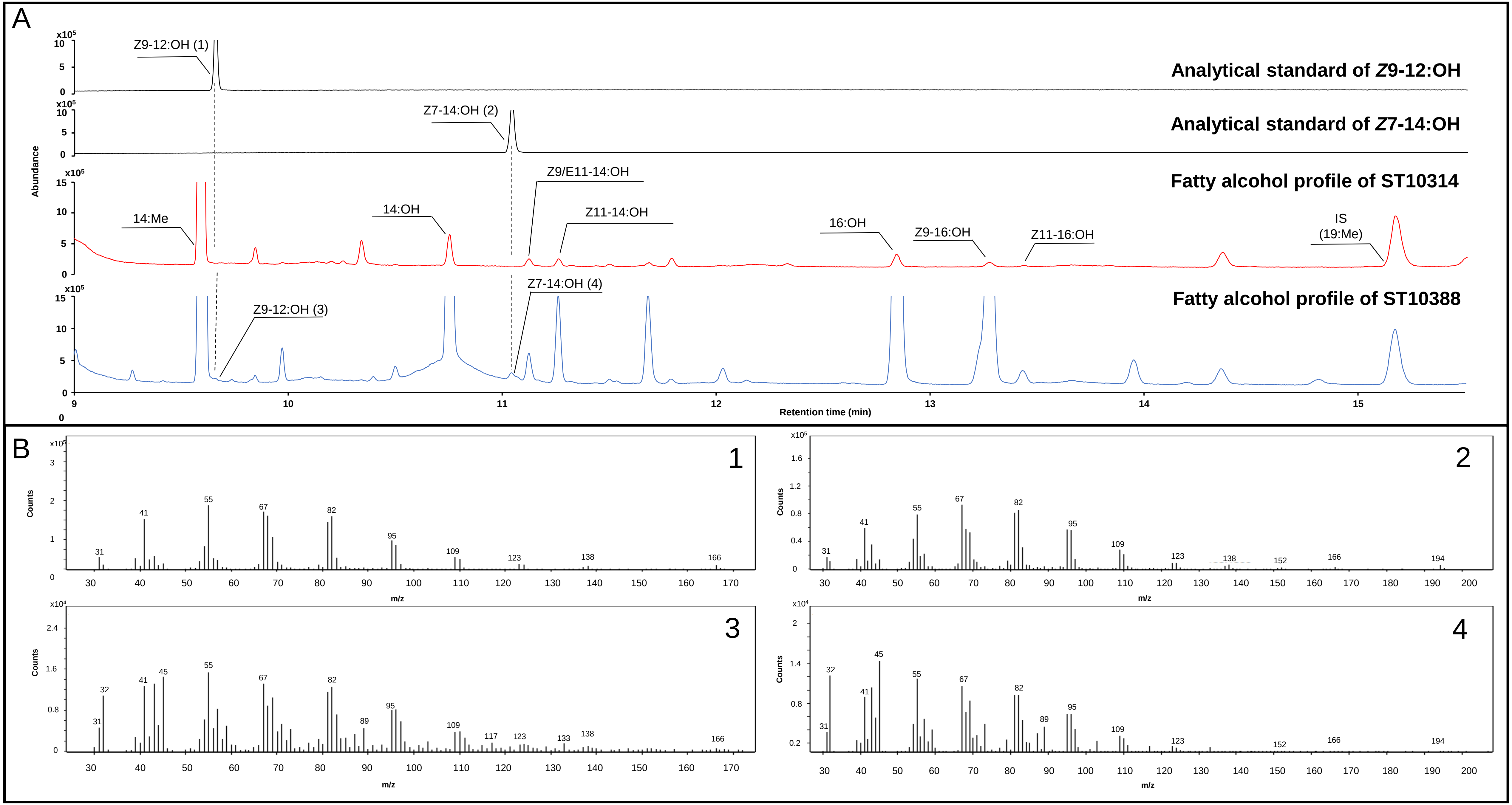

Figure S6.A. GC-MS chromatograms of Z9-12:OH and Z7-14:OH reference standards together with fatty alcohol profiles of ST10314 (red) and ST10388 (blue). B. Mass spectra of Z9-12:OH reference standard (1) and Z7-14:OH reference standard together with mass spactra of biologically produced Z9-12:OH (3) and Z7-14:OH (4). Cultivation media was supplemented with 0.4% (v/v) of 14:Me.

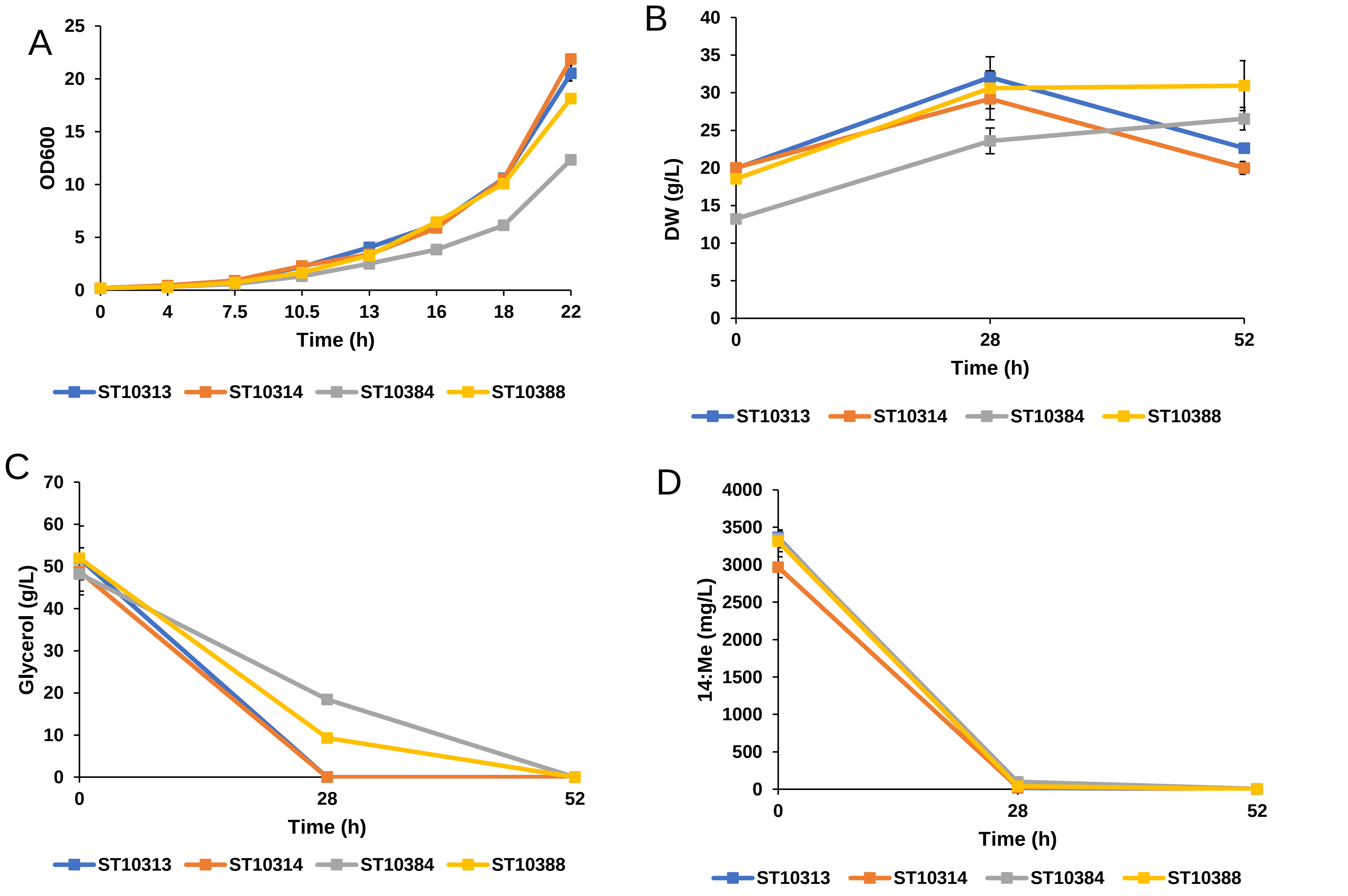

Figure S7. Growth characteristics and utilization of glycerol and 14:Me by *Y. Lipolytica* strains ST10313, ST10314, ST10384, ST10388 in shake flasks. A. Growth curves during the first 22h in YPG media. B. Dry weight (DW) measurements at 0, 28 and 52h after media exchange. C. Glycerol measurements at 0, 28 and 52h after media exchange. D. 14:Me measurements at 0, 28 and 52h after media exchange. Error bars represent standard deviations. OD measurements were performed in technical dublicates. DW, glycerol and 14:Me measurements were performed in technical triplicates.

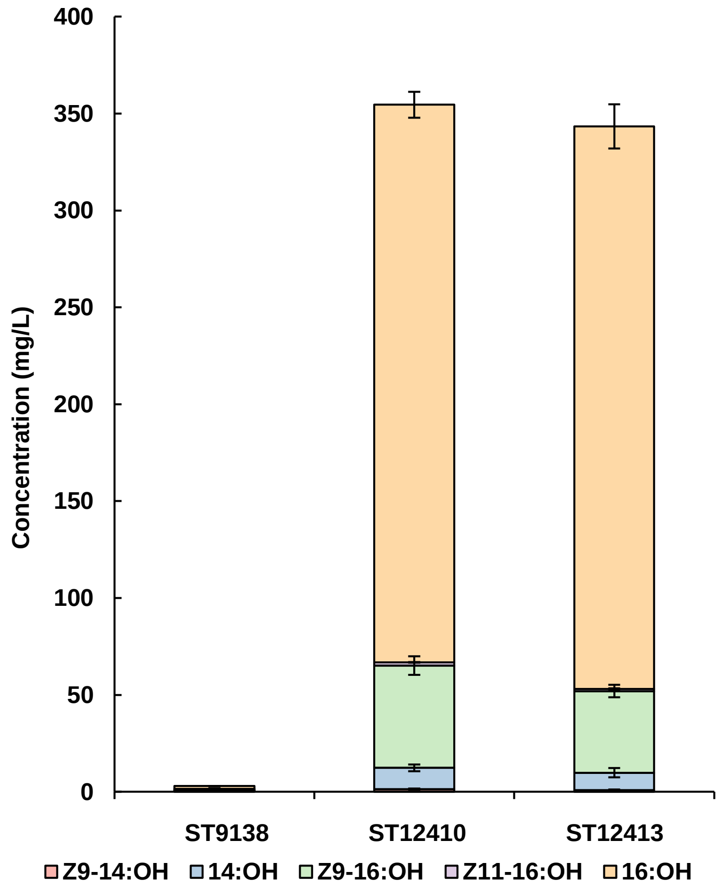

Figure S8. Fatty alcohol profiles of the *Y. lipolytica* strains expressing HarFAR-hrGFP-Per2 (ST12410) and HarFAR-hrGFP (ST12413) fusion proteins. ST9138 is the strain that does not have FAR. Error bars represent standard deviations from three technical replicates
